# Beyond Mortality: Exploring the Influence of Plant Phenolics on Modulating Ferroptosis—A Systematic Review

**DOI:** 10.3390/antiox13030334

**Published:** 2024-03-10

**Authors:** Nemanja Živanović, Marija Lesjak, Nataša Simin, Surjit K. S. Srai

**Affiliations:** 1 Department of Chemistry, Biochemistry and Environmental Protection, Faculty of Sciences, University of Novi Sad, Trg Dositeja Obradovica 3, 21000 Novi Sad, Serbia; nemanja.zivanovic@dh.uns.ac.rs (N.Ž.); marija.lesjak@dh.uns.ac.rs (M.L.); natasa.simin@dh.uns.ac.rs (N.S.); 2 Research Department of Structural and Molecular Biology, Division of Biosciences, University College London, Darwin Building, Gower Street, London WC1E 6BT, UK

**Keywords:** ferroptosis, polyphenols, ferroptosis initiation, ferroptosis inhibition

## Abstract

Ferroptosis is a recently discovered type of programmed cell death that is mechanistically different from other types of programmed cell death such as apoptosis, necroptosis, and autophagy. It is characterized by the accumulation of intracellular iron, overproduction of reactive oxygen species, depletion of glutathione, and extensive lipid peroxidation of lipids in the cell membrane. It was discovered that ferroptosis is interconnected with many diseases, such as neurodegenerative diseases, ischemia/reperfusion injury, cancer, and chronic kidney disease. Polyphenols, plant secondary metabolites known for many bioactivities, are being extensively researched in the context of their influence on ferroptosis which resulted in a great number of publications showing the need for a systematic review. In this review, an extensive literature search was performed. Databases (Scopus, Web of Science, PubMed, ScienceDirect, Springer) were searched in the time span from 2017 to November 2023, using the keyword “ferroptosis” alone and in combination with “flavonoid”, “phenolic acid”, “stilbene”, “coumarin”, “anthraquinone”, and “chalcone”; after the selection of studies, we had 311 papers and 143 phenolic compounds. In total, 53 compounds showed the ability to induce ferroptosis, and 110 compounds were able to inhibit ferroptosis, and out of those compounds, 20 showed both abilities depending on the model system. The most researched compounds are shikonin, curcumin, quercetin, resveratrol, and baicalin. The most common modes of action are in the modulation of the Nrf2/GPX4 and Nrf2/HO-1 axis and the modulation of iron metabolism.

## 1. Introduction

Cell death is a fundamental process present in all living organisms, executed through various mechanisms. For a long time, cells were thought to be removed in two ways—regulated cell death, called apoptosis, and unregulated cell death, called necrosis [1]. Recently, many processes of controlled cell death have been discovered, such as intrinsic apoptosis, extrinsic apoptosis, anoikis, autophagy-dependent cell death, programmed cell death (physiological cell death), entotic cell death, necrosis, necroptosis, oxytosis/ferroptosis, pyroptosis, paraptosis, parthanatos, oxeiptosis, and NETosis. These processes of cell death are characterized by different methods of induction and the involvement of specific signaling pathways [2].

Apoptosis, often referred to as controlled or programmed cell death, is a highly regulated process that plays a crucial role in various physiological and pathological situations. It can be triggered by different factors, including significant cellular damage or the activation of specific receptors on the cell membrane, all characterized by the activation of caspases [3]. Ferroptosis, on the other hand, is a mechanism of cell death that occurs due to significant oxidative damage to cells. It is characterized by the depletion of glutathione, increased levels of free intracellular iron, and elevated lipid peroxidation in the cell membrane [4,5]. Ferroptosis is believed to play a role in various pathological conditions, including neurodegenerative diseases, chronic kidney disease, cardiovascular diseases, as well as ischemia/reperfusion injury [6,7,8]. Conversely, inducing ferroptosis is considered a potential therapeutic approach for addressing liver fibrosis and cancer—conditions characterized by disrupted normal cell activity leading to pathology [9,10].

Plants generate a countless number of compounds through secondary metabolism, exhibiting protective and hormonal functions within the plant itself. Interestingly, these compounds also possess high bioactivity in animals and humans. These characteristics have made plants integral to traditional medicinal practices spanning centuries. In contemporary times, the advancement of scientific knowledge has given rise to new disciplines like rational phytotherapy. The primary objective of such fields is to formulate safe and scientifically substantiated medicines derived from plants. Consequently, there is a substantial and growing interest in the research of medicinal plants and their bioactive compounds [11].

### 1.1. Apoptosis

The term apoptosis was proposed in 1972, in a paper written by Kerr, Wyllie, and Currie. They described apoptosis as programmed cell death that is complementary to mitosis. Apoptosis is defined by two phases: the first phase is characterized by the formation of spheroid apoptotic bodies and the second by their phagocytosis and finally degradation by other cells [12]. There are two distinct apoptotic pathways—intrinsic and extrinsic. Both pathways culminate with the activation of caspases, which are proteases responsible for cleaving numerous vital proteins within the cell. This process leads to the manifestation of apoptotic cell morphology and eventual cell death, typically occurring within a time span of minutes to hours.

**Figure 1:**
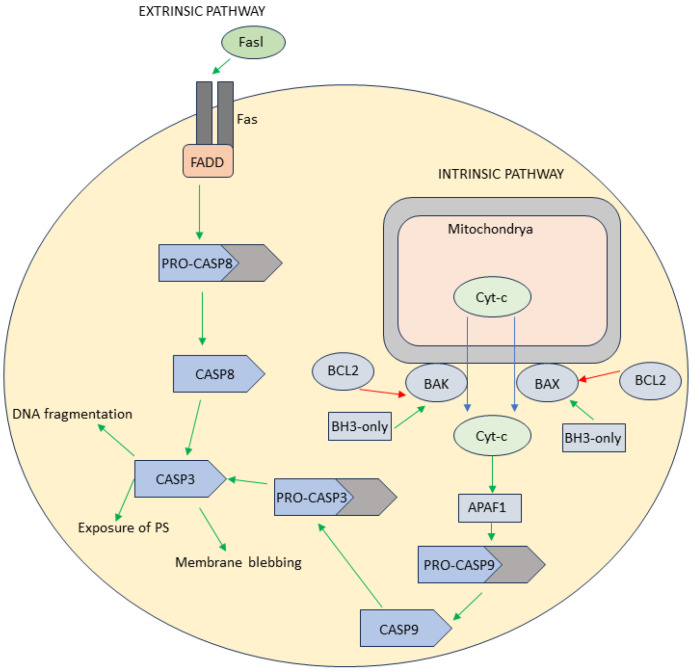
Mechanism of apoptosis initiation. The activation of the intrinsic pathway triggers the release of Cyt-c from the mitochondrial lumen into the cytosol, facilitated by a pore formed by the proteins BAK and BAX. This event induces the formation of a protein complex involving APAF1 and pro-CASP9, ultimately leading to the release of active CASP9. Subsequently, CASP9 activates CASP3. On the other hand, the extrinsic pathway is initiated by the binding of a ligand to its receptor, as illustrated in the scheme depicting the binding of Fasl to Fas. This binding activates a cascade that culminates in the activation of CASP8, which in turn activates CASP3. The activation of CASP3 serves as a pivotal point of no return, leading to the exposure of PS on the outer layer of the cell membrane, membrane blebbing, and the fragmentation of DNA in the nucleus.

The intrinsic pathway is activated when there is significant damage caused by different types of stress inside the cell, such as DNA damage, endoplasmic stress, and lack of growth factors. Signaling pathways activated under stress can disturb the equilibrium between the BCL2 family of proteins with anti-apoptotic functions and proapoptotic proteins from family BH3-only. Equilibrium can be pushed towards the proapoptotic side by overexpression of BH3-only proteins, while simultaneously posttranslational modifications and proteolytic processing of BCL2 proteins occurs, and excess of BH3-only proteins leads to the initiation of apoptosis. BH3-only proteins bind to BCL2 proteins, as well as to BAK and BAX proteins which are necessary for apoptosis. Following the activation of BAX and BAK, pores in the outer mitochondrial membrane are formed. That process is termed mitochondrial outer membrane permeabilization, and it results in the outflow of many molecules from the mitochondrial intermembrane space, among which is cytochrome c (Cyt-c). Cyt-c is necessary for the activation of the apoptotic protease-activating factor 1 (APAF1). APAF1 contains the CARD (caspase recruitment domain) which interacts with the CARD of pro-caspase 9 (pro-CASP9) forming apoptosome. Inside this complex, apoptosis initiator caspase 9 (CASP9) is formed from pro-caspase 9, and subsequently, it leads to the activation of caspases 3 and 7 (CASP3 and CASP7) (Figure 1) [1,13].

The initiation of the extrinsic pathway occurs external to the cell, triggered by environmental conditions that signify the necessity for the cell to undergo apoptosis. The extrinsic pathway is activated by the binding of particular ligands to cell surface death receptors and consists of three signaling pathways: tumor necrosis factor receptor (TNFR), TNF-related apoptosis-inducing ligand (TRAIL), and factor-associated suicide (Fas)/Fas ligand (FasL) pathways [14]. When FasL binds to the Fas receptor, it leads to activation of the signaling pathway. The Fas-associated death domain (FADD) activates pro-caspase 8 (pro-CASP8), after which, active caspase 8 (CASP8) activates CASP3 and CASP7 (Figure 1) [15].

Activation of CASP3 and CASP7, by both intrinsic and/or extrinsic pathways, marks a point of no return and the cell has no choice but to undergo suicide (Figure 1). These caspases are involved in processes that define apoptosis, such as the fragmentation of DNA, exposure of phosphatidylserine (PS) on the outer lipid bilayer, blebbing of the cell membrane, and formation of apoptotic bodies. CASP3 promotes DNA fragmentation through the proteolytic inactivation of DNA fragmentation factor subunit alpha (DFFA or ICAD), which results in the release of active DFFB (also known as CAD). CASP3 is also involved in the externalization of PS through the activation of enzymes called phospholipid scramblases. Additionally, CASP3 inhibits enzymes—flippases—involved in PS internalization [3]. The mechanism of apoptosis initiation is shown in Figure 1.

### 1.2. Ferroptosis

Ferroptosis, initially proposed by Dr. Brent R. Stockwell and collaborators in 2012, represents a pivotal advancement in cell biology. Its discovery emerged from a comprehensive investigation spanning several years. Between 2001 and 2003, Dr. Stockwell’s laboratory delved into the exploration of small molecules exhibiting HRAS^V12^-selective lethality. This intensive research culminated in the identification of a compound named erastin, characterized by its capacity to induce cell death via a non-apoptotic mechanism. Subsequent investigations further elucidated the properties of erastin and led to the discovery of RAS-selective lethal-3 (RSL3). Both compounds demonstrated the ability to trigger cell death, not through apoptosis, but rather via an iron-oxidative stress-dependent pathway.

These groundbreaking findings, along with complementary discoveries by other research groups, collectively defined ferroptosis as a novel form of regulated cell death. Ferroptosis is distinguished by its dependence on intracellular iron accumulation and the production of reactive oxygen species (ROS). Moreover, they identified it as a cell death mechanism that is morphologically, biochemically, and genetically distinct from previously known cell death mechanisms [4,5]. This paradigm shift in our understanding of cellular demise underscores the intricate interplay between iron metabolism, oxidative stress, and cell fate regulation. The elucidation of ferroptosis mechanisms holds profound implications for various fields, including cancer biology, neurodegenerative diseases, and therapeutic development.

#### 1.2.1. Lipid Metabolism and Ferroptosis

**Figure 2:**
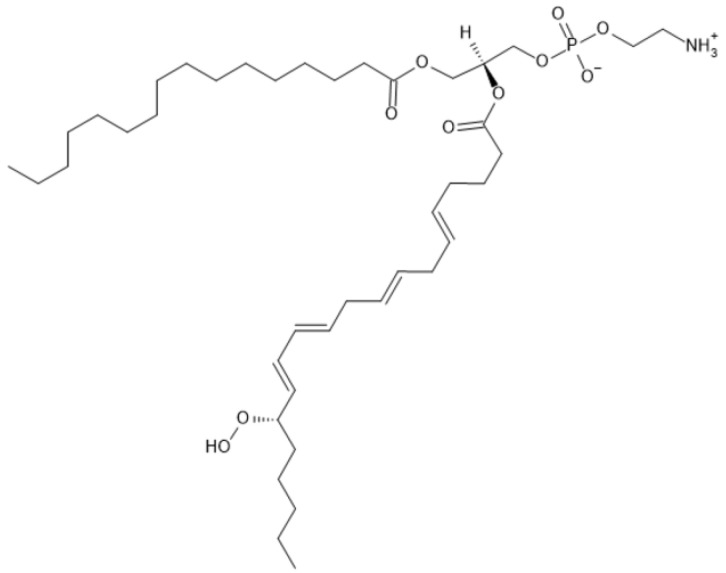
Structure of PE-peroxide containing AA—it is a product of ALOX12/15 enzymes, is very unstable, and can initiate a lipid peroxidation chain reaction leading to damage of cell membrane.

**Figure 3:**
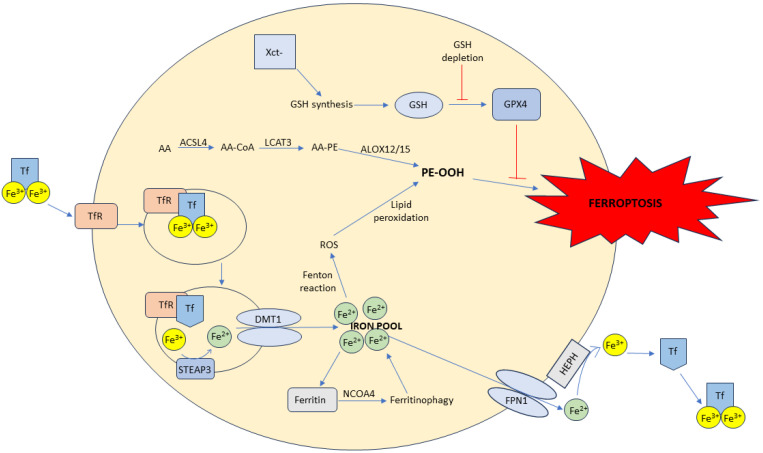
Mechanism of initiation of ferroptosis. The Xct- system is vital for preventing ferroptosis by supplying cystine for GSH synthesis, used by GPX4 to neutralize lipid radicals, as marled with red line. Under intense oxidative stress, depleted GSH and blocked Xct- system hinders sufficient GSH production, leaving the cell vulnerable to oxidative damage. ACSL4 synthesizes phospholipids with unsaturated fatty acids, like AA, prone to oxidation by ALOX12/15 enzymes, inducing lipid peroxidation in the cell membrane. Accumulated free iron in the cytosol generates reactive hydroxyl radicals through the Fenton reaction, triggering highly reactive lipid peroxides and initiating lipid peroxidation in the cell membrane. Iron levels are tightly regulated; in the blood, Fe^3+^ is transported bound to Tf. Cells with TfR receptors internalize the Tf-iron complex, releasing iron through DMT1. Ferritin stores iron inside cells, shielding them from pro-oxidant free iron. Ferritinophagy releases iron into the cytosol. Excess iron exits cells through FPN1, oxidizes to Fe^3+^, and binds to Tf. Processes inhibiting Xct-, GPX4, or causing free iron accumulation, along with increased synthesis of unsaturated fatty acid phospholipids and activation of ALOX12/15, can promote ferroptosis.

Ferroptosis is characterized by the accumulation of free iron in cells which leads to high production of ROS and the subsequent lipid peroxidation of plasma membrane lipids. In the following text, mechanisms of ferroptosis induction are explained in detail. In ferroptosis, oxidized polyunsaturated fatty acids (PUFAs), from membrane phospholipids, play an important role. ACSL4 (acyl-CoA synthetase long-chain family member 4) is an enzyme that catalyzes the biosynthesis of PUFA-CoA, among others also from arachidonic acid (AA) and adrenic acid (AdA). PUFA-CoA are then used for the synthesis of phosphatidylethanolamines (PE) containing AA (PE-AA) and AdA (PE-AdA), catalyzed by LPCAT3 (lysophosphatidylcholine acyltransferase 3). These PEs are substrates for ALOX (arachidonic acid lipoxygenases) enzymes (ALOX15 and ALOX12), which catalyze peroxidation reactions in which PE-peroxides are formed (PE-OOH, Figure 2). PE-OOH are very unstable and can initiate lipid peroxidation which can damage the lipid bilayer (Figure 3) [16].

#### 1.2.2. Iron Metabolism and Ferroptosis

Iron uptake by cells (apart from intestinal cells) occurs via transferrin receptors (TfR) which are positioned on the cell membrane. These receptors bind transferrin (Tf), the iron transporter in plasma, after which they are internalized as endosomes. In endosomes, under acidic conditions, Fe^3+^ is released from the Tf-TfR complex. After release, Fe^3+^ is reduced to Fe^2+^ by the STEAP3 (six-transmembrane epithelial antigen of prostate 3), and exported in the cytosol by DMT1 (divalent metal transporter 1). In the cytosol, free Fe^2+^ can initiate the Fenton reaction, which is prevented by binding to ferritin. Ferritin is a protein that stores excess iron in the form of Fe^3+^. PCBP1 and PCBP2 (poly rC-binding protein) are cytoplasmic proteins that are involved in the transportation of iron to ferritin. NCOA4 (nuclear receptor coactivator 4) mediates ferritinophagy, the autophagy degradation of ferritin in lysosomes, resulting in the release of iron into the cytosol (Figure 3) [17]. It has been shown that, in the first phases of ferroptosis, ferritinophagy plays a crucial role and that inhibitors of ferritinophagy can slow down the ferroptosis. However, in later stages, this inhibition does not exhibit a significant effect [18].

Iron can be exported from the cell, such as in enterocytes, hepatocytes, and macrophages, into the bloodstream by ferroportin-1 (FPN1). FPN1 exports iron as Fe^2+^, which is immediately oxidized to Fe^3+^ by hephaestin (HEPH) or ceruloplasmin. Hephaestin is expressed in enterocytes, while ceruloplasmin is expressed in macrophages and hepatocytes [19]. Once oxidized to Fe^3+^, iron can be bound to Tf (Figure 3). Hepcidin is a peptide hormone secreted by the liver that plays a key role in the systemic regulation of iron metabolism. Hepcidin binds to FPN1, and the complex is internalized in the cell and degraded. Higher levels of hepcidin will lead to the inhibition of iron export and subsequent iron accumulation [20].

#### 1.2.3. Role of Xct-/GPX4 Axis

The Xct- transportation system is responsible for the supply of cystine inside the cell, which is necessary for the synthesis of glutathione (GSH). This system consists of a regulatory unit (SLC3A2) and a transportation unit (SLC7A11). The availability of cystine determines the rate of GSH synthesis. GSH is a substrate for glutathione peroxidase 4 (GPX4), the enzyme that is responsible for the reduction of lipid peroxides, such as PE-OOH, to lipid alcohol, PE-OH. Xct-/GSH/GPX4 is the axis that is responsible for the inhibition of ferroptosis. Inhibition of the Xct- system and/or GPX4 results in the initiation of ferroptosis, and vice versa (Figure 3) [21]. Blocking the Xct- system also results in elevated intracellular free iron levels, heightening the cell’s vulnerability to ferroptosis. Specifically, labile iron forms a complex with GSH and binds to PCBP1. Depletion of GSH inhibits iron binding to PCBP1, leading to the accumulation of free iron, which can generate ROS through the Fenton reaction [4,22].

#### 1.2.4. GCH1/DHFR/BH4 Axis

**Figure 4:**
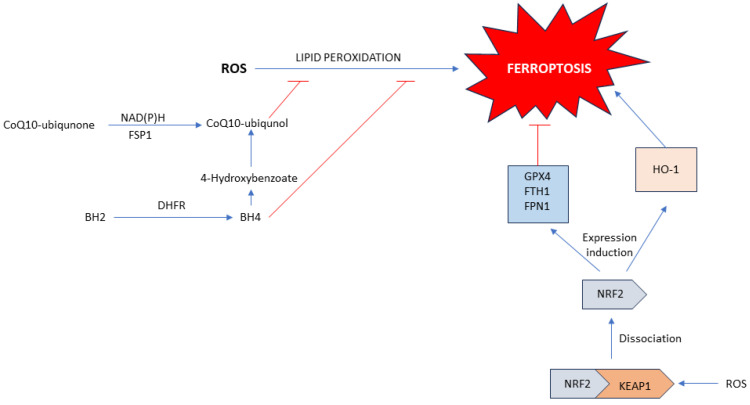
DHFR/BH4, FSP1/NAD(P)H/CoQ10, and Nrf2 pathways. CoQ10 and BH4 antioxidants help to neutralize harmful lipid radicals and prevent lipid peroxidation. The red line indicates the neutralization of reactive species, which consequently inhibits lipid peroxidation. FSP1 is an enzyme that turns inactive CoQ10 into its active form, ubiquinol. DHFR is another enzyme that converts dihydrobiopterine (BH2) to BH4, acting as an antioxidant and aiding in CoQ10 synthesis. Keap1 inhibits the release of Nrf2. During oxidative stress, Nrf2 is freed from Keap1, moves to the nucleus, and boosts the expression of proteins that inhibit ferroptosis, as marked with red line.

Likewise, GCH1 (GTP cyclohydrolase-1) is the rate-limiting enzyme for the synthesis of tetrahydrobiopterin (BH4). Lower levels of BH4 indicate higher sensitivity to ferroptosis (Figure 4). BH4 is not only a reducing agent in cells but also serves as a cofactor of many enzymes. BH4 is necessary for the conversion of phenylalanine into tyrosine, which is necessary for the production of 4-OH-benzoate—a precursor of CoQ_10_ [23]. DHFR (dihydrofolate reductase) is the enzyme responsible for the regeneration of BH4. Inhibition of this enzyme, along with inhibitors of GPX4 can cause ferroptosis. The GCH1/DHFR/BH4 axis can neutralize lipid peroxides and lipid radicals, protecting cells from ferroptosis (Figure 4) [20].

#### 1.2.5. FSP1/NAD(P)H/CoQ10 Axis

FSP1/NAD(P)H/CoQ10 is a GPX4-independent system for cell protection against ferroptosis (Figure 4). Namely, FSP1 (ferroptosis suppressor protein 1) belongs to the quinone oxidoreductase NDH-2 family and is associated with the plasma membrane. It reduces ubiquinone to ubiquinol via NAD(P)H and is included in the synthesis of CoQ10-ubiquinol. Namely, CoQ10 is located in membranes where it serves as an antioxidant. It can neutralize lipid radicals and protect cells from ferroptosis. Moreover, FSP1 can also regenerate α-tocopherol (vitamin E), another potent antioxidant in the plasma membrane [20].

#### 1.2.6. Keap1/Nrf2 Axis

Nuclear factor erythroid 2-related factor 2 (Nrf2, Figure 4) is a transcriptional factor that under normal conditions is bound to Kelch-like ECH-associated protein 1 (Keap1) and degraded through the ubiquitin-proteasomal pathway. Inhibition of Keap1 leads to the release and activation of Nrf2 [24]. Nrf2 translocates to the nucleus, interacts with antioxidant-responsive elements (AREs) and promotes the expression of many proteins. Some targets of Nrf2 are responsible for the prevention of ferroptosis, such as GPX4, ferritin light and heavy chains (FTL, FTH1), and FPN1. On the other hand, Nrf2 promotes the expression of proteins that can enhance ferroptosis such as heme-oxygenase 1 (HO-1), which is responsible for the degradation of heme from hemoglobin, products of this reaction are biliverdin, CO, and Fe^2+^ [25].

#### 1.2.7. Role of p53 in Ferroptosis

Tumor suppressor protein p53 plays an important role in the suppression of tumor formation and growth by regulating the cell division and response of the cell to different types of stress. p53 can inhibit the expression of SLC7A11, making cells more susceptible to ferroptosis. p53 P47S polymorphism leads to increased levels of GSH and the inhibition of ferroptosis, probably due to the inability to suppress the expression of SLC7A11. p53 can activate the expression of SAT1 (Spermidine/spermine N1-acetyltransferase 1), which leads to the induction of the expression of ALOX15 which can lead to ferroptosis and tumor suppression. The exact mechanism of activation of this p53/SAT1/ALOX15 metabolic pathway, and the induction of ferroptosis through it, is still not fully elucidated. Additionally, p53 can translocate in mitochondria in hepatic stellate cells and cause the accumulation of redox-active iron and induction of ferroptosis. On the other hand, p53 can also act as a ferroptosis suppressor. Namely, p53 regulates the localization of DPP4 (dipeptidyl peptidase 4). The proposed mechanism is that depletion of p53 leads to decreased levels of DPP4 in the nucleus, which in turn leads to membrane-associated DPP4- binding to NOX1 (NADPH oxidase 1), lipid peroxidation, and ferroptosis. Another pro-survival effect of p53 is through p21 also known as CDKN1A (cyclin-dependent kinase inhibitor 1A). It has been shown that the p53-dependent expression of p21 and the production of GSH can delay ferroptosis, but this mechanism is not fully elucidated. All this highlights the importance of p53 in tumor progression and shows that p53 can be an important ferroptosis modulator in cancer cells and the loss of function of p53 can make cancer cells more resistant to ferroptosis, thus promoting cancer growth [26,27].

### 1.3. Plant Secondary Metabolites

Numerous plants have been historically employed in traditional medicinal practices over the centuries. Presently, there exists a substantial interest in the utilization of medicinal plants and harnessing plant secondary metabolites for medicinal purposes. This has led to the development of fields such as rational phytotherapy and pharmacognosy, as well as the design and approval of several methods of phytotherapeutics by national health regulatory bodies [11].

Plants produce a wide range of secondary metabolites. Their concentration is higher when plants are under stress, suggesting that they have a protective role. They are divided into three big groups, alkaloids, phenolics (PC), and terpenoids, and show many bioactivities that can be beneficial for humans and animals, such as antioxidant, anti-inflammatory, antihypertensive, anti-aging, and insulin-sensitizing properties [28,29].

**Figure 5:**
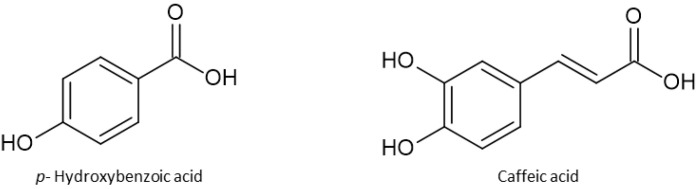
Structures of most common phenolic acids.

Phenolics or polyphenols represent a class of numerous compounds that possess one or more phenolic group in their structure. They can be divided into subclasses such as phenolic acids, flavonoids, tannins, coumarins, stilbenes, and lignans. Phenolic acids are polyphenols that are derived from hydroxybenzoic and hydroxycinnamic acids, and they differ by the number and position of hydroxy and methoxy groups. The most common benzoic acids are p-hydroxybenzoic, vanillic, and protocatechuic, while the most common hydroxycinnamic acids are caffeic, ferulic, and p-coumaric (Figure 5). Hydroxycinnamic acids are often found in esters with quinic acid called chlorogenic acids, while the most common are esters with caffeic acid. They possess antioxidant, anti-inflammatory, and antitumor activities [30].

**Figure 6:**
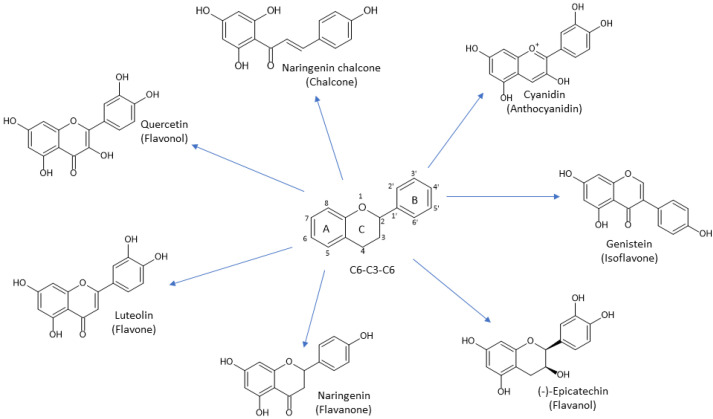
Structures of seven flavonoid subclasses’ skeletons and some of their representatives.

Flavonoids are one of the biggest classes of plant polyphenols. They have a C6-C3-C6 skeleton made of the three rings denoted as A, B, and C (Figure 6). They are divided into seven subclasses: flavanones, flavanols, anthocyanidins, flavones, flavonols, and chalcones. Flavonols have a 2-phenyl-chromen-4-one backbone and hydroxyl group bound at position C3, and representatives of this subclass are quercetin, rutin, and kaempferol. Flavones differ from flavonols due to their lack of hydroxyl group at position C3; apigenin and luteolin are the most common members of this subclass. Flavanones are also termed dihydroflavones because they do not have double bonds between C2 and C3. They are present in citrus fruits, with hesperidin, naringin, and eriodyctiol as representatives. Flavanols, known as flavan-3-ols, do not have double bond between C2 and C3 and a keto group at C4 but possess a hydroxyl group at C3. They are present as monomers or as polymers making proanthocyanidins. They are present in different fruits with (+)-catechin and (−)-epicatechin as the most common ones. Isoflavones are characterized by a 3-phenyl-chromen-4-on backbone, and thanks to this structural characteristic they are similar to animal estrogens and show affinity for estrogen receptors. They are known as phytoestrogens, and daidzein and genistein are members of this flavonoid subclass. Anthocyanidins are characterized by having flavylium cation as their backbone which makes them unstable and present as anthocyanins. In plants, they are pigmented with red, purple, and blue colors in flowers and fruits. The most common anthocyanidins are cyanidin, malvidin, delfinidin, and pelargonidin. Chalcones are a specific subclass of flavonoids because they have an open C-ring. They are precursors for the biosynthesis of flavonoids and isoflavonoids. Flavonoids have strong antioxidant activity and other beneficial properties for human health such as anti-hypertensive, cardioprotective, anti-inflammatory, anti-cancer, and neuroprotective properties [31].

**Figure 7:**
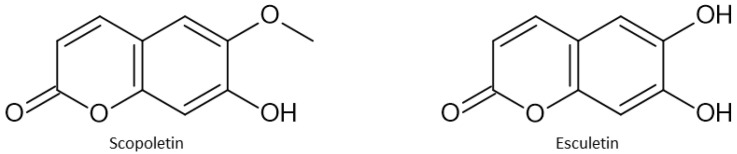
Structures of simple coumarin representatives.

Coumarins are a class of polyphenols that, at the core of their structure, have a 2H-chromene-2-on structure. They are divided into several classes: simple coumarins (including hydroxycoumarins—scopoletin, esculetin), furanocoumarins (methoxsalen), pyranocoumarins (samidin, braylin), benzocoumarins (urolithin A and B), phenylcoumarins, and biscoumarins (Figure 7). They possess numerous biological properties such as anticoagulant, antioxidant, antiangiogenic, anticancer, and antibacterial properties [32].

**Figure 8:**
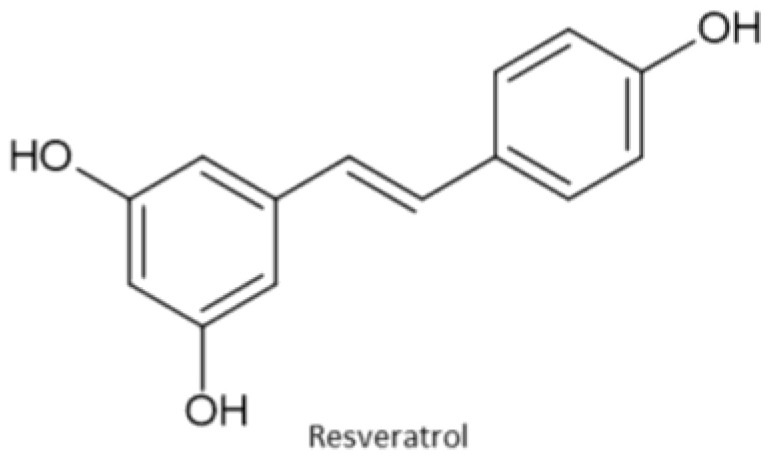
Structure of stilbene representative—resveratrol.

Stilbenes are polyphenols with a diphenylethylene (C6-C2-C6) structure. In plants, they are produced as a defense mechanism against infections and UV radiation [33]. A representative of stilbenes is resveratrol (Figure 8). It is a polyphenolic stilbene with a double bond connecting two phenolic rings. Under UV radiation, it can undergo geometric isomerization. The trans-isomer is more abundant than the cis-isomer, and also more bioactive. Resveratrol possesses many activities that can be beneficial for humans such as cardioprotective, antioxidant, anti-inflammatory, anti-fungal, anti-viral, and other activities [34].

Tannins are polymers, and they are divided into hydrolyzable and non-hydrolyzable condensed tannins. Hydrolysable tannins are composed of ellagic and gallic acids with a sugar core. On the other hand, condensed tannins are composed of flavonoids (flavan-3-ol and flavan-3,4-diol). Tannins have anticancerogenic, antiviral, anticancer, and antioxidant activities [35].

As can be seen from the above text, plants produce a plethora of polyphenolic compounds with many positive impacts on human health. In recent years, many papers have been published with evidence that plant secondary metabolites can modulate ferroptosis. In this paper, we will try to give some overview of those results and insights for future research in this field.

## 2. Methodology

This review work was undertaken under the guidance of the Preferred Reporting Items for Systematic Review and Meta-analysis (PRISMA) [36].

### 2.1. Search Strategy

A comprehensive search of multiple databases was performed. The searched databases were Web of Science, PubMed, ScienceDirect, Scopus, and Springer in the time span from 2017 to November 2023. The search was limited to research articles written in English and the keyword used was “ferroptosis”, in combination with “polyphenol”, “flavonoid”, “phenolic acid”, “coumarin”, “stilbene”, “chalcone”, “anthraquinone”.

### 2.2. Inclusion Criteria

Studies investigating the influence of pure, naturally occurring plant polyphenols on ferroptosis in any type of cells, tissue, or organism were considered. From each paper, the following information was extracted: investigated compound, study design, proposed mechanism of action, first author, and year of publication.

### 2.3. Exclusion Criteria

Studies investigating the influence of semi-synthetic or synthetic compounds not naturally occurring in plants; studies investigating extracts; and studies with in silico investigation without experimental confirmation of activity toward ferroptosis were excluded.

## 3. Results and Discussion

**Figure 9:**
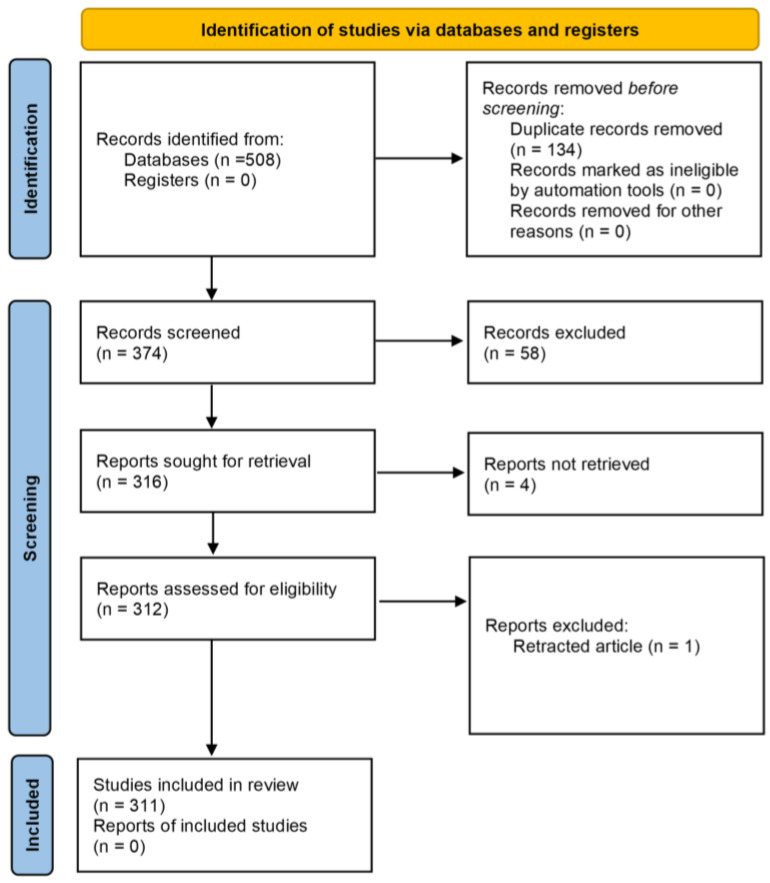
Prisma flow diagram. Illustrates search strategy and selection process. Selected databases were searched using keywords which resulted in a collection of 508 papers. After the removal of duplicates, papers outside of scope and not retrieved papers, 311 papers were included in this review.

The selection process according to PRISMA guidelines is shown in Figure 9 and the PRISMA checklists are given in the Supplementary Materials (Tables S1 and S2) The research protocol for this study has been registered at INPLASY (registration number INPLASY202410104, https://doi.org/10.37766/inplasy2024.1.0104, accessed on 7 March 2024). A comprehensive search of databases resulted in the identification of 508 papers. After removing 134 duplicates and 58 studies that did not meet the inclusion criteria or that were not relevant, 4 papers were not retrieved and 312 studies were fully read. In total, one paper, previously included, was removed because of a retraction notice. In the end, 311 papers were included in this review. From the included papers, data were extracted and summarized in tables. Data extraction was performed by two independent researchers (N.Z. and M.L.).

### 3.1. Studying Influence on Ferroptosis

Research on the ferroptosis influence on cells can be carried out in vitro and in vivo. In vitro research is carried out on many different cell lines, mostly cancer cells [37,38,39,40]. In vivo studies are carried out on animals, most commonly mice and rats, but other species can also be used like gerbils and zebrafish, as shown in Table 1 and Table 2 [41,42,43,44,45].

#### 3.1.1. Studies on Ferroptosis Inhibition

According to this review, research into the inhibition of ferroptosis is conducted both at the cellular and animal levels. In cell studies, ferroptosis is initiated, followed by the analysis of the inhibitory potential of the tested compounds. Ferroptosis initiation is induced by incubating cells with substances like erastin (Xct- system inhibitor), RSL3 (GPX4 inhibitor), hemin, or others [46,47]. Standard ferroptosis inhibitors, such as liproxstatin-1 (Lip-1) and ferrostatin-1 (Fer-1), are employed for comparison with the treatments under examination [47]. In animal studies, chemicals or surgical procedures are employed to induce ferroptosis, followed by the administration of phytochemicals to assess their anti-ferroptosis activity. Parameters examined include GSH, oxidized GSH, malondialdehyde (MDA), free cellular iron levels, and the expression of genes and proteins involved in the regulation of ferroptosis, such as GPX4, HO-1, Nrf2, FTH1 [7,48,49,50].

#### 3.1.2. Studies on Ferroptosis Induction

For ferroptosis initiation/promotion studies, similarly to inhibition studies, cells are grown under the usual conditions until treatment with examined compounds. Parameters that are being examined are the same as for inhibition studies, levels of GSH, MDA, free iron, ROS, expression of GPX4, HO-1, FTH-1, Nrf2, among others. The inclusion of standard ferroptosis inhibitors in the treatment serves as an additional validation of ferroptosis initiation. Ferroptosis initiators (erastin and RSL3) can be used as a standard for evaluating the potency of the examined compound as a ferroptosis initiator. In ferroptosis initiation studies, animals are often inoculated with cancer cells in order to induce tumor formation, in which ferroptosis induction can be a potential treatment. Animals are usually divided into groups that receive treatment with the examined compound and control which receives saline or buffer solution. In addition to the standard parameters examined in tissues, these studies also track changes in tumor size [41,51,52,53,54].

### 3.2. Polyphenols as Potential Therapeutic Agents in Diverse Diseases via Ferroptosis Modulation

The involvement of ferroptosis in diverse diseases positions it as a promising target for therapeutic intervention. Plant secondary metabolites, particularly polyphenols, exhibit significant promise in the potential development of novel treatments centered on the modulation of ferroptosis.

#### 3.2.1. Ferroptosis as Potential Target for Cancer Treatment

Cancer stands out as a major concern in contemporary society and medicine research. Existing therapies lack the desired selectivity, causing significant harm to healthy tissues and organs, thereby posing substantial challenges for patients. The emerging concept of ferroptosis presents a novel and promising path in cancer treatment. Considerable research has been dedicated to exploring plant secondary metabolites, especially polyphenols, as inducers of ferroptosis, thereby serving as potential anticancer drugs. Numerous cancer cell lines undergo scrutiny in this research, complemented by a substantial body of in vivo studies utilizing tumor xenograft models [54,55,56,57].

Specifically, a variety of polyphenols have been verified as potential anti-cancer agents, and notably, curcumin, whose potency to promote ferroptosis consequently demonstrated significant anti-cancer effects across various cancer cell types. It has the ability to reduce the expression of GPX4, SLC7A11, and FPN1, while simultaneously increasing the expression of ACSL4. This modulation resulted in the accumulation of iron, overproduction of ROS, and cell death through ferroptosis [10,51,58,59,60,61]. Furthermore, amentoflavone [62,63,64], baicalin [54,55,65], erianin [37,41,57,66,67], gambogenic acid [68], auriculasin [69], wogonin [70], quercetin [71,72], shikonin [73,74], scoparone [75], osthole [76], also have shown to be able to inhibit cancer growth though the induction of ferroptosis. All of this suggests the potential for developing novel cancer treatments grounded in natural products acting as ferroptosis inducers.

#### 3.2.2. Ferroptosis as Potential Target for Diabetes Treatment

Diabetes is a significant burden of contemporary society as another prevalent disease. It is defined by the body’s incapacity to regulate normal blood sugar levels, stemming from either insulin resistance or insufficient insulin production. Additionally, diabetes can contribute to the development of other conditions, such as diabetic nephropathy, cardiomyopathy, and neuropathies [77,78,79]. Interestingly, polyphenols quercetin, rhein, and glabridin have been shown to attenuate diabetic nephropathy through inhibition of ferroptosis. Quercetin acted through the activation of the Nrf2/HO-1 pathway, rhein acted via the Rac1/NOX1/β-catenin pathway, while glabridin acted on the VEGF/Act/Erk pathway [78,80,81]. Furthermore, curcumin showed the ability to ameliorate diabetic cardiomyopathy by inhibiting ferroptosis. It was able to activate the Nrf2 pathway, raising levels of GPX4 and HO-1 and finally inhibiting ferroptosis caused by high glucose levels [82]. It is crucial to underscore that certain polyphenols exhibit a dual effect on ferroptosis, acting as both inhibitors and enhancers of this process. For instance, curcumin exemplifies this phenomenon. In cancer research, curcumin has demonstrated the ability to promote ferroptosis in cancer cells, thereby inducing cell death. Conversely, in diabetes, curcumin has been shown to inhibit ferroptosis, leading to cell revival. It is conceivable that curcumin operates through cell-specific mechanisms concerning ferroptosis. While these findings may be confusing, ongoing research endeavors aim to elucidate these phenomena further. Dihydromyricetin, ferulic acid, and naringin protected neurons from death and prevented cognitive impairment associated with diabetes and a high-fat diet. Dihydromyricetin acted as an inhibitor of the JNK-inflammatory pathway, oxidative stress, and lipid peroxidation, and caused downregulation of ACSL4 and upregulation of GPX4 expression [83]. On the other side, ferulic acid and naringin are proven to activate the Nrf2/GPX4 axis which leads to the inhibition of ferroptosis and neuroprotection [77,84].

#### 3.2.3. Ferroptosis as Potential Target in Treatment of Neurodegenerative Diseases

Neurodegenerative diseases such as Parkinson’s and Alzheimer’s are caused by neuronal degeneration, accompanied by synaptic dysfunction, inflammation, oxidative stress, and the accumulation of proteins such as synuclein, Aβ, and tau proteins. Studies have shown that neuron death in these diseases is at least partially caused by iron metabolism imbalance and ferroptosis. Polyphenols have shown potential to at least partially improve and alleviate these conditions [85,86,87]. Specifically, it was shown that chrysophanol protected neurons from ferroptosis in Alzheimer’s disease by decreasing ROS levels, inhibiting lipid peroxidation, and increasing GPX4 expression [86]. Eriodictyol showed potential in the prevention and treatment of neurodegenerative disease thanks to its ability to guard neurons from oxidative stress and ferroptosis through activation of the Nrf2 pathway [88,89]. Echinatin [90], gastrodin [47,91,92] icariin [93], isoforthiaside [85], quercetin [94,95], salidroside [96,97], sennoside A [87], and tetrahydroxy stilbene glycoside [98] have been shown to prevent neurodegeneration and ameliorate neurodegenerative diseases, such as Alzheimer’s, through activation of the Nrf2 signalling pathway, which protects against oxidative stress and ferroptosis. Unlike them, silibinin protects against Alzheimer’s disease and neurodegeneration by inhibiting ferroptosis and inflammation through modulation of the STING pathway [99].

#### 3.2.4. Ferroptosis as Potential Target in Traumatic Brain Injury Treatment

A traumatic brain injury can cause damage to blood vessels and brain tissues leading to haemorrhage and neuron death. Similar to an intracranial haemorrhage after a traumatic brain injury, a subarachnoid haemorrhage, which can be caused by a ruptured aneurism, leads to damage of surrounding brain tissues. The neuronal damage is at least partially caused by the degradation of released haemoglobin by HO-1, which leads to the accumulation of free iron, oxidative stress, lipid peroxidation, and neuronal death by different mechanisms, including ferroptosis [46,100,101,102]. There is no treatment for these conditions, but some polyphenols show promising results in this regard. Baicalein and baicalin have been shown to also alleviate brain injury through the inhibition of ferroptosis. They were able to increase the expression of GPX4 and SLC7A11 which led to protection against oxidative damage [46,103,104]. Puerarin is another compound that showed potential in the treatment of a brain injury caused by an intracerebral haemorrhage. It activated the AMPK/PGC1α/Nrf2 pathway, which was followed by the alleviation of iron accumulation, lipid peroxidation inhibition, an increase in the expression of GPX4, and a decrease in the expression of ACSL4 [101].

#### 3.2.5. Ischemia/Reperfusion Injury Treatment by Targeting Ferroptosis

Ischemia/reperfusion injury arises when blood flow is interrupted, leading to hypoxia, followed by the restoration of blood flow and reoxygenation. This phenomenon can be manifested in the myocardium, brain, and various other organs. It is common for this to occur during major surgeries, myocardial infarction, stroke, and organ transplantations [105]. It is marked by elevated production of ROS, diminished levels of antioxidants, inflammation, and cell death. New discoveries suggest that ferroptosis plays a role in ischemia/reperfusion injury, offering a fresh approach to treating this condition. Plant secondary metabolites, especially polyphenols, have exhibited encouraging outcomes as potential therapeutic agents in this context. In vivo studies revealed that isorhamnetin and apigenin-7-O-(6″-p-coumaroyl)-glucoside can attenuate ischemia/reperfusion injury by inhibiting ferroptosis [106,107]. Galangin was able to attenuate ischemia/reperfusion injury through inhibition of ferroptosis by activating Nrf2/GPX4, SLC7A11/GPX4, and PI3K/AKT/CREB signaling pathways [44,108,109]. Resveratrol also showed potential to attenuate ischemia/reperfusion injury through anti-ferroptotic activity [110,111,112]. Some other compounds that displayed promising results for treatment of ischemia/reperfusion injuries were rhein [113], vitexin [114], naringenin [115], loureirin C [116], caffeic acid [117], baicalein [118,119], luteolin [119], kaempferol [120], and others. The predominant mechanism underlying this phenomenon involves the upregulation of GPX4 and SLC7A11 expression, along with the modulation of iron metabolism.

#### 3.2.6. Ferroptosis as Potential Target in Treatment of Liver Diseases

The liver serves as a central hub in the body’s defense system, playing a crucial role in metabolizing and detoxifying various chemicals and drugs. Some substances, such as acrylamide and acetaminophen, have been identified as potential contributors to liver injury [121,122,123,124]. Nevertheless, certain polyphenols have demonstrated significant potential in mitigating liver injury. For instance, salidroside has been shown to alleviate acetaminophen-induced acute liver injury. Its mechanism involves inhibiting endoplasmic reticulum stress-dependent ferroptosis through the activation of AMPK/SIRT1 and the inhibition of the ATF4/CHAC1 axis [124]. Similarly, kaempferol was proven effective in ameliorating acetaminophen-induced liver injury. It achieved this by activating the Nrf2 pathway, leading to the upregulation of GPX4 and SOD2 expression, the reduction of ACSL4 expression, alleviation of intracellular iron accumulation, and ultimately the inhibition of ferroptosis [125]. Additionally, epigallocatechin-gallate has demonstrated its ability to alleviate liver injury induced by a high-fat diet. The accumulation of triglycerides in the liver due to a high-fat diet renders the organ susceptible to damage from oxidative stress, inflammation-induced lipid peroxidation, and ferroptosis. Epigallocatechin-gallate protects the liver by mitigating iron accumulation, increasing the expression of GPX4, decreasing the expression of ACSL4 and COX2, and ultimately inhibiting ferroptosis [126].

Liver fibrosis is a prevalent condition associated with numerous chronic liver diseases, marked by the excessive production and build up of extracellular matrix proteins, including α-smooth muscle actin and collagens. Hepatic stellate cells play a pivotal role in this process, as they undergo activation and contribute to the synthesis of matrix proteins. Research has indicated that specifically targeting and inducing the death of these cells can mitigate fibrosis, with ferroptosis emerging as a potential targeted mechanism for this purpose [127,128]. Chrysophanol has expressed some potential to attenuate liver fibrosis by removing activated hepatic stellate cells through the induction of ferroptosis through the downregulation of GPX4 and SLC7A11 expression [128]. Danshensu, isoliquiritigenin, and wogonoside are phenolic compounds with the proven potential to alleviate liver fibrosis through induction of ferroptosis in hepatic stellate cells. They downregulated the expression of GPX4 and SLC7A11, and promoted ROS production and lipid peroxidation which leads to cell death through ferroptosis [9,127,129].

#### 3.2.7. Acute Lung Injury Treatment through Ferroptosis Modulation

Acute lung injury can be caused by different mechanisms such as infections, non-chest traumas, massive blood transfusions, acute pancreatitis, and prolonged oxygen supplementation during hospitalization. It is characterized by the production of inflammatory mediators, increased permeability of the barrier between the capillary endothelium and alveolar epithelial cells and pulmonary oedema. It has been shown that non-apoptotic cell death mechanisms, such as ferroptosis, are implicated in acute lung injury. The most common treatments for acute lung injury are glucocorticoids, which come with serious side effects. Ferroptosis inhibition is seen as a new potential target for the development of new therapeutics [130,131,132]. Chicoric acid was able to alleviate LPS-induced lung injury in mice thanks to its anti-inflammatory and anti-oxidative activities. It reduced the production of inflammatory cytokines and ROS, inhibited lipid peroxidation, increased levels of GSH and activated the Nrf2/HO-1 pathway [133]. Ferulic acid and Naringenin are other polyphenolics with a proven ability to alleviate acute lung injury through activation of the Nrf2/HO-1 signaling pathway and subsequent inhibition of ferroptosis [134,135]. Also, quercetin alleviated lung injury through ferroptosis inhibition by activating the Sirt1/Nrf2/GPX4 pathway [130].

Significantly, as described before, both the induction and inhibition of ferroptosis by polyphenols have demonstrated therapeutic benefits in different pathological states. As this review aims to provide the latest data on the potential use of polyphenols in the context of ferroptosis, it endeavors to contribute to the exploration of these compounds as potential therapeutics for the future. By presenting up-to-date information, this review seeks to elucidate the nuanced role of polyphenols in modulating ferroptosis and their potential applications in addressing various pathological conditions. The multifaceted effects of polyphenols on ferroptosis pave the way for a comprehensive understanding of their therapeutic potential, marking them as promising candidates for future medical interventions. Specifically, Table 1 presents a compilation of polyphenols capable of inducing ferroptosis, accompanied by their verified mechanisms of action, the model systems in which these effects were examined, and corresponding references. Additionally, Table 2 provides parallel information on polyphenols identified as inhibitors of ferroptosis. These tables serve as comprehensive up-to-date references, offering valuable insights into the diverse actions of polyphenols in modulating ferroptosis.

**Table 1:** List of polyphenols as inducers and enhancers of ferroptosis, verified mechanisms of their action, the model systems in which these effects were examined, and corresponding references.

Phenolics	Proposed Mechanism	Model System in Which Effect Was Examined		Reference
		In Vitro	In Vivo	
Agrimonolide	Increases ROS and Fe^2+^ levels and downregulates GPX4 and SLC7A11	A2780 and SKOV-3 cells	SKOV-3 cells xenograft model in BALB/c mice	[136]
Alloimperatorin	Accumulation of Fe, ROS, and MDA, decreased expression of SLC7A11 and GPX4 with increased expression of Keap1	Breast cancer cells	/	[137]
Amentoflavone	Induces ferroptosis through miR-496/ATF2 axis	GSE-1, AGS and HGC-27 cells	AGS cells xenograft model in BALB/c nude mice	[62]
	Promotes ferroptosis by activation of ROS/AMPK/mTOR pathway	ESC and KLE cell lines	/	[63]
	Increased levels of iron, lipid ROS, and MDA, depletion of GSH	U251 and U373 cells	U251 cells xenograft model in BALB/c nude mice	[64]
Apigenin	Causes iron accumulation, lipid peroxidation, GSH depletion, downregulates expression of SLC7A11 and GPX4, upregulates expression of p62, HO-1, and ferritin	Human Ishikawa cells	Ishikawa cells xenograft model in BALB/c nude mice	[52]
	Nanocomposites Fe_2_O_3_/Fe_3_O_4_@mSiO_2_ loaded with apigenin induce ferroptosis by increasing ROS production and downregulation of expression of GPX4, FTH1 and upregulation of expression of COX2 and p53	A549 and HUVECs	/	[138]
Auriculasin	Increases levels of Fe^2+^ and MDA, and induces generation of ROS, promotion of expression of Keap1 and AIFM	HCT116 and SW480 cells	/	[69]
Baicalin	Promotes Fe^2+^ accumulation, ROS production, lipid peroxidation, depletes GSH, decreases expression of GPX4 and Xct- through upregulated degradation of Nrf2	MG63, 143B, hBMSC	MG63 xenograft model in BALB/c nude mice	[54]
	Promotes ROS production, enhances mRNA expression of TfR1, NOX1 and COX2, suppresses mRNA expression of GPX4, FTH1, and FTL	AGS and SGC-7901 cells	/	[55]
	Induces ferroptosis through downregulation of FTH1	Bladder cancer cells 5637 and KU-19-19 cells	KU-19-19 cells xenograft model in mice	[65]
Bavachin	Increases Fe^2+^ through increased DMT1 and TfR and decreased FTH and FTL expression, depletes GSH and enhances ROS production and MDA, modulates STAT3/p53/SLC7A11 axis	MG63 and HOS cell lines	/	[139]
Bilobetin	Promotes iron accumulation, ROS production and upregulates p53 expression	HCT116, HT29, RKO and LOVO cells	HCT116 xenograft model in BALB/c mice	[140]
Chrysin	Induces ferritinophagy-dependent ferroptosis	PANC-1, Capan-2, BxPC-3, AsPC-1 and HPDE6-C7 cells	PANC-1 xenograft model in BALB/c nude mice	[141]
Chrysophanol	Accumulation of ROS, downregulation of GPX4 and SLC7A11	HSC-T6 cell line	/	[128]
Curcumin	Induces ferroptosis through GSH/GPX4 and FSP1/CoQ10-NADH pathways	A549 cells	A549 cells xenograft model in NOD/SCID mice	[10]
	Promotes iron accumulation and lipid peroxidation, upregulates expression of HO-1, and downregulates expression of GPX4	Nthy-ori-3-1, FTC-133 and FTC-238 cells	/	[58]
	Induces ROS production and downregulates expression of GPX4 and FSP1	SW480 and HCT116 cells	/	[56]
	Induces ferroptosis through upregulation of SLC1A5	MDA-MB-453 and MCF-7	MCF-7 cells xenograft model in BALB/c nude mice	[61]
	Promotes iron accumulation and lipid peroxidation, downregulates GSH, GPX4, and SLC7A11, activates PI3K/Akt/mTOR pathway	HCT-8 cells	/	[59]
	Depletion of GSH, accumulation of iron and ROS, lipid peroxidation	A549 and H1299 cells	Lewis lung carcinoma model in C57BL/6 mice	[51]
	Induces ferroptosis through ADAMTS18	Sunitinib-resistant A498 and 786-O cells	/	[60]
	Accumulation of intracellular iron, ROS, LOOH, MDA, and upregulation of HO-1	MCF-7 cells	/	[142]
Danshensu	Promotes ROS production and downregulates expression of GPX4 and SLC7A11	LX-2 and T6 cells	/	[129]
3,5-dicaffeoylquinic acid	Induces ferroptosis and mitochondrial dysfunction through ROS/AMPK/mTOR pathway	HCT116 and SW480 cells	/	[143]
4,4′-dimethoxychalcone	Promotes iron accumulation, lipid peroxidation, and activates Keap1/Nrf2/HO-1 pathway	A549 cells and 786-O cells	/	[144]
Diplacone	Promotes lipid peroxidation ATF3 expression	A549, NK-92 and K562 cells	/	[145]
Emodin	Downregulates Notch1/Nrf2/GPX4 pathway	NRK-52E cells	Emodin high-dose induced nephrotoxicity in mice	[146]
Erianin	Ferrostatin-1 inhibits cytotoxicity of erianin suggesting activation of ferroptosis	A549 and H1299 cells	A549 cells xenograft model in nude mice	[66]
	Promotes accumulation of iron and ROS production, induces lipid peroxidation and downregulates expression of GPX4, FTH1, and ferritin	LoVo, HCT116, DLD1, HCT8, SW480, SW620, HT29, Caco2, MC38 cells	HCT116-luc cells subcutaneous and liver metastasis models in BALB/c nude mice	[37]
	Promotes iron accumulation and lipid peroxidation and downregulates expression of GPX4, upregulates expression of ALOX12, p53, and METTL3	CD44+/CD105+ HuRCSCs	Xenograft model in BALB/C^nu/nu^ mice	[57]
	ROS accumulation, GSH depletion, lipid peroxidation, and downregulation of FTH1, GPX4, HO-1, Xct-/SLC7A11, and Nrf2	RT4 and KU-19-19 cells	KU-19-19 cells xenograft model in BALB/c nude mice	[41]
	Induces ferroptosis through activation of Ca^2+^/CaM signaling pathway	H460 and H1299 cells	H460 cells xenograft model in BALB/c nude mice	[67]
Eriocitrin	Downregulates Nrf2/SLC7A11/GPX4 axis	A549 and H1299 cells	/	[147]
Eriodictyol	Downregulates expression of SLC7A11 and GPX4, downregulates Nrf2/HO-1/NQO1 pathway	A2780 and CaoV3 cells	CaoV3 cell xenograft model in BALB/c mice	[148]
Esculetin	Promotes iron accumulation and lipid peroxidation through NCOA4 mediated ferritinophagy	HUH7 and HCCLM3 cells	HUH7 cells xenograft model in BALB/c mice	[149]
Ferulic acid	Upregulates ACSL4 and HO-1 and downregulates SLC7A11 and GPX4	TE-4 and EC-1 cells	/	[150]
Gallic acid	Downregulates expression of GPX4 and SLC7A11 by inhibiting Wnt/β-catenin pathway	HepG2 cells	/	[151]
	Potential ferroptosis inducer, and increased ROS and MDA production, reduced activity of GPX.	MDA-MB-231, MCF10A, and A375 cells	/	[152]
	Cell death triggered by gallic acid can be prevented by ferroptosis inhibitor DFO	HeLa, SH-SY5Y, and H446 cells	/	[153]
Gambogenic acid	Activates p53/SLC7A11/GPX4 axis	HOS and 143B cells	143B cells xenograft model in athymic nude mice	[154]
	Activates AMPKα/SLC7A11/GPX4 pathway	FHC, HCT116, HT29, DLD-1, HCT115 and COLO320DM cells	HCT116 cells xenograft model in BALB/c mice	[40]
	Downregulates NEAT1 and induces ferroptosis through SLC7A11/GPX4 axis	A375, B16, B16F10 and A2058 cells	B16F10 xenograft model in C57BL/6 mice	[68]
	Induces ferroptosis through p53/SLC7A11/GPX4 axis	A375 and A2058 cells	/	[155]
6-gingerol	Enhanced production of ROS, depletion of GSH, downregulated expression of Nrf2 and GPX4	LNCaP, DU145, and PC3 cells	/	[156]
	Upregulates expression of NCOA4 and TfR1, and downregulates expression of GPX4 and FTH1	A549 cells	A549 cells xenograft model in BALB/c mice	[157]
Ginkgetin	Promotes iron accumulation, ROS production, and upregulates p53 expression	HCT116, HT29, RKO and LOVO cells	HCT116 xenograft model in BALB/c mice	[140]
	Enhances cytotoxicity of DDP through induction of ferroptosis, increases levels of free iron, production of ROS, and lipid peroxidation, lowers GSH/GSSG ratio, reduces protein levels of SLC7A11 and GPX4, inhibits Nrf2/HO-1 axis	A549, NCI-H460, SPC-A-1 cells	NSCLC xenograft model in nude mouse	[158]
Honokiol	Promotes lipid peroxidation and upregulates expression of HO-1	THP-1, U-937, and SMK-1 cells	/	[159]
	Increases levels of iron and ROS, and downregulates expression of GPX4	SW48, HT29, LS174T, HCT116, HCT8, RKO, and SW480 cells	RKO cells xenograft model in BALB/c mice	[160]
Icariin	Induces autophagy and ferroptosis through modulation of miR-7/mTOR/SREBP1 pathway	RWPE-1, DU145, and PC-3 cells	/	[161]
Icariside II	Upregulates miR-324-3p which downregulates GPX4	ACHN, A498, 786-O, Caki-1, and 293T cells	Caki-1 cells xenograft model in BALB/c mice	[162]
Isoginkgetin	Promotes iron accumulation, ROS production, and upregulates p53 expression	HCT116, HT29, RKO, and LOVO cells	HCT116 xenograft model in BALB/c mice	[140]
Isoliquiritigenin	Upregulates expression of HO-1, and downregulates expression of GPX4	SGC996, NOZ, and L-2F7 cells	NOZ cells xenograft model in BALB/c mice	[163]
	Downregulates GPX4, and upregulates TfR and DMT1 expression	HSC-T6 cells	CCl_4_-induced liver-fibrosis model in C57BL/6 mice and 0.06% TA-induced liver fibrosis model in zebrafish	[9]
Isoliquiritin	Promotes iron accumulation and downregulates expression of GPX4 and SLC7A11	MDA-MB-231 and MCF-7 cells	BALB/c mice	[164]
Isoorientin	Inhibits SIRT6/Nrf2/GPX4 signaling pathway	A549 cells	A549 cells xenograft model in BALB/c mice	[165]
Isoquercitrin	Downregulates expression of GPX4, HO-1 and SLC7A11	CNE1 and HNE1 cells	CNE1 cells xenograft model in BALB/c mice	[166]
Luteolin	Promotes expression of ACSL4, and downregulates expression of GPX4, SLC7A11, FTH1, and FTL1	RWPE-1, DU145, PC-3, and LNcaP cells	PC-3 cells xenograft model in BALB/c mice	[167]
	Acts synergistically with erastin, and upregulates expression of HIC1 which results in downregulated expression of GPX4	HCT116, SW480, and NCM460 cells	HCT116 cells xenograft model in BALB/c mice	[168]
Lysionotin	Promotes degradation of Nrf2	HCT116, SW480, HIEC, and NCM460 cells	HCT116 cells xenograft model in athymic nude mice	[169]
Nobiletin	Increases levels of iron, MDA, and ROS, decreases levels of GSH and GPX4, enhances the expression of Keap1, and inhibits the expression of Nrf2 and HO-1	SK-MEL-28	/	[170]
Osthole	Downregulates expression of GPX4 and SLC7A11, and upregulates expression of HO-1 and NCOA4	HCT116, SW480, and MC38 cells	HCT116 cells xenograft model in C57BL/6J mice	[76]
Puerarin	Promotes autophagy-dependent ferroptosis through upregulation of NCOA4	HT-29 cells	/	[171]
Plumbagin	Promotes ROS production and GPX4 degradation	HepG2 and Hep3B cells	/	[172]
Quercetin	Downregulates expression of GPX4, and upregulates expression of SLC7A11 and TfR1	HEC-1-A cells	/	[173]
	Triggers the release of iron through degradation of ferritin, enhances the production of ROS and lipid peroxidation, increases expression of SLC27A4 and decreases expression of GPX1	HepG2, Hep3B, MDA-MB-231, HCT116 cell lines	/	[71]
Resveratrol	Promotes ferroptosis through Hsa-miR-335-5p/NFS1/GPX4 pathway	AML-193 and OCI-AML-3 cells	/	[174]
	Upregulates expression of ACSL4, and downregulates expression of GPX4 and SLC7A11	Human plasma and lung tissues, PBMCs and H520 cells	/	[175]
Rhamnazin	Downregulates expression of GPX4	HCC Huh7 and SMMC-7721 cell	/	[176]
Robustaflavone A	Accumulation of ROS, downregulation of Nedd4, and upregulation of VDAC2	MCF-7 cells	/	[177]
Rotenone	Promotes iron accumulation, promotes expression of ACSL4, COX2, GPX4 and SLC7A11	Primary neuron culture	Intracerebral hemorrhage model in ICR mice	[178]
	Upregulates expression of Keap1, p53, and COX2, and downregulates expression of GPX4, SLC7A11, and FTH1	NE-4C cells	/	[179]
	Inhibits Keap1/Nrf2/SLC7A11/GPX4 axis	BV-2 cells	/	[180]
S-3′-hydroxy-7′, 2′, 4′-trimethoxyisoxane	Inhibits Nrf2/HO-1 signaling pathway	SGC-7901, A549, H460, SW480, BEL-7402, HeLa, HBE, and MCF-7 cells	A549 cells xenograft model in nude mice	[181]
Scoparone	Activaties the ROS/JNK/SP1/ACSL4 axis	NHBE, A549, H1299, and PC-9 cells	/	[75]
Shikonin	Inhibits Nrf2 signaling pathway	HTR-8/SVneo cells and villous tissue of women suffering from TP and from women with normal pregnancies	/	[182]
	Downregulates expression of GPX4, and upregulates expression of TfR1, NCOA4, HO-1, and shows synergism with cisplatin	A2780, SKOV3, OVCAR4, A2780/DDP, SKOV3/DDP, and OVCAR4/DDP cells	A2780/DPP cells xenograft model in BALB/c nude mice	[73]
	Downregulates expression of GPX4 and promotes expression of ATF3	SBC-2 and H69 cells	SBC-2 cells xenograft model in nude mice	[74]
	Induces GOT1-dependent ferritinophagy	RPMI 8226 and U266 cells	RPMI 8226 cells xenograft model in Nod-SCID mice	[183]
	Shikonin-Fe(III) nanoparticles induce iron accumulation, GSH depletion, production of ROS, increase lipid peroxidation and downregulate GPX4 expression	4T1 and L929 cells	4T1 xenograft model in BALB/c mice	[184]
Theaflavin-3,3′-Digallate	Induces iron accumulation and lipid peroxidation, downregulates expression of GPX4 and FTH1	HOS, MG63, and hFOB1.19 cells	HOS cells xenograft model in BALB/c mice	[185]
2,3,5,4′-tetrahydroxystilbene	Upregulates expression of ACSL4 and downregulates expression of GPX4	DLD-1, HT-29, HCT-116	HT-29 xenograft model in severe combined immunodeficient (SCID) mice	[53]
Tiliroside	Downregulates expression of GPX4 and SLC7A11 and upregulates expression of HO-1	BT-549, MDA-MB-468, SK-BR-3, MCF-7, and MCF-10A cells	BT-549 cells xenograft model in BALB/c mice	[186]
	Promotes ubiquitination of Nrf2, and downregulates expression of GPX4 and FTH1	HepG2, Hep3B SMMC-7721, and L02 cells	HepG2 xenograft model in BALB/c mice	[187]
Typhaneoside	Increases ROS production, and lipid peroxidation and reduces GSH and GPX. Promotes autophagy and autophagic degradation of ferritin	Kas-1, HL60, NB4, K562 cells	HL60 cells xenograft model in BALB/c mice	[38]
Wogonin	Inhibits Nrf2/GPX4 pathway	AsPC-1, PANC-1, and HPDE6-C7 cells	PANC-1 cells xenograft model in BALB/c mice	[70]
Wogonoside	Modulates SOCS1/p53/SLC7A11 pathway to induce ferroptosis	HSC-t6, AML12, and RAW 264.7 cells	CCl_4_-induced liver fibrosis model in C57BL/6 mice	[127]

**Table 2:** List of polyphenols with inhibitory activity on ferroptosis, verified mechanisms of their action, the model systems in which these effects were examined, and corresponding references.

Phenolics	Proposed Mechanism	Model System in Which Effect Was Examined		Reference
		In Vitro	In Vivo	
Acacetin	Upregulates expression of GPX4 and downregulates expression of ACSL4	HepG2 cells	High-fat-diet-induced lipid accumulation in liver in C57BL/6 mice	[188]
Acetyl zingerone	Activates Nrf2/HO-1 signaling pathway	Primary chondrocytes	Knee osteoarthritis model in C57BL/6J mice	[189]
Aloe-emodin	Complexes Fe^2+^, modulates intracellular iron metabolism, increases expression of Nrf2, GPX4, and SLC7A11	H9c2 cells	/	[190]
Anacardic acid	Downregulates expression of TfR1, and upregulates expression of GPX4	/	Freeney free-fall impact model-induced traumatic brain injury in ICR mice	[102]
Anhydroxysafflor yellow B	Reduces ROS, 4-HNE, and MDA production, reduces Fe^2+^ levels, increases GSH/GSSG ratio, and upregulates expression of GPX4 and SLC7A11	PC12 cells	/	[191]
Apigenin	Decreases production of ROS and MDA, upregulates activity of SOD, and improves GSH/GSSG ratio, lowers level of Fe^2+^	AML12 cells	/	[192]
Apigenin-7-O-(-6″-p-coumaroyl)-glucoside	Reduces the level of ROS and Fe^2+^, inhibits HO-1 and MAO-B	Human umbilical vein endothelial cell line (HUVECs)	Model of intestinal ischemia/reperfusion injury in CL57BL/6J mice	[107]
Astringin	Antioxidant activity	bmMSCs	/	[193]
Avicularin	Activates Nrf2/HO-1/GPX4 pathway	RAW 246.7 and HepG2 cells	LPS/D-GalN-induced acute liver failure model in C57BL/6 mice	[194]
Baicalein	Decreases iron accumulation and lipid peroxidation, upregulates expression of GPX4 and SLC7A11, downregulates expression of ALOX15	IEC-6 cells	CPT-11-induced gastrointestinal dysfunction in Wistar rats	[195]
	Decreases levels of ROS and ALOX15	/	Cardiac arrest model in SD rats	[196]
	Inhibits ferroptosis through a decrease in p53 acetylation level by elevating SIRT1	HK2 cells	Polymyxin B-induced acute kidney injury model in C57BL/6 mice	[197]
	Alleviates ferroptosis through AMPK/Nrf2/HO-1 axis	Primary human and mouse chondrocyte	Surgery-induced osteoarthritis model in C57BL/6J mice	[198]
	Decreases levels of ROS, MDA, and iron, and restores protein levels of GPX4	H9c2 cells and primary cardiomyocytes from neonatal SD rats	Langerdorff perfusion system for ischemia/reperfusion model on isolated hearts from SD rats	[119]
	Modulates GPX4/ACSL4/ACSL3 axis	HT22 cells	tMCAO-induced cerebral ischemia/reperfusion injury model in C57BL/6 mice	[118]
	Decreases iron and MDA levels, and suppresses expression of HMOX1 and FPN1, and increases expression of GPX4 and SLC7A11	THP1 cells and human macrophages isolated from endometrium peritoneal fluid	/	[199]
	Inhibits NF-κB pathway, and activates Nrf2/HO-1 pathway	HepG2 cells	CCl_4_-induced acute liver injury in C57BL/6 mice	[121]
	Upregulates GPX4, and downregulates TFR1.	HEM-1 cells	/	[200]
	Suppression of lipid peroxidation, lover levels of 4-HNE, upregulation of GPX4, and downregulation of LOX12/15	HT22 cells	FeCl3-induced post-traumatic epilepsy disorder in C57/BL6 mice	[103]
Baicalin	Enhanced expression of GPX4 and SLC7A11, decreased expression of DMT1	PC12 cells and primary cortical neurons	Type IV collagenase-induced intracerebral hemorrhage model in C57BL/6 mice	[46]
	Reduces ROS production, and promotes SOD activity and expression of GPX4, lowers iron levels through modulation of iron uptake, storage, and ferritinophagy	H9C2 cells	Myocardial ischemia/reperfusion injury model in male Sprague-Dawley rats	[201]
	Decreased levels of Fe^2+^, MDA and ROS, increased level of GSH and upregulation of GPX4	Primary rat neurons	Subarachnoid hemorrhage model in Sprague-Dawley rats	[104]
Biochanin A	Downregulates TfR1, upregulates FPN1, and activates Nrf2/system xc-/GPX4 pathway	Primary chondrocytes isolated from C57BL/6 mice	Iron-dextran-induced iron overload and surgery-induced knee osteoarthritis in C57BL/6 mice	[202]
Caffeic acid	Downregulates expression of ACSL4 and TfR1 and activates Nrf2 pathway	SK-N-SH cells	pMCAO-induced cerebral ischemia/reperfusion injury	[117]
Cannabinol	Inhibits ROS production and lipid peroxidation, increases expression of Nrf2, HO-1, SOD2, GPX4	HT22, SH-SY5Y, BV2 cells	/	[203]
Calycosin	Downregulates expression of ACSL4 and TfR1, and upregulates expression of FTH1 and GPX4	PC12 cells	tMCAO/R model in SD rats	[204]
	Upregulates expression of GPX4, and downregulates expression of NCOA4	HK-2 cells	Db/db mice and db/m mice	[205]
Cardamonin	Increases expression of SLC7A11, GPX4, p53, and decreases expression of iNOS and COX2	Primary chondrocytes isolated from SD rats	Osteoarthritis model in SD rats	[48]
Carthamin yellow	Downregulates expression of ACSL4 and TfR1, and upregulates expression of GPX4 and FTH1	/	MCAO-induced cerebral ischemia/reperfusion model in SD rats	[206]
Chebulagic acid	Inhibits ferroptosis through ROS scavenging and iron chelation	Bone marrow-derived mesenchymal stem cells isolated from SD rats	/	[207]
Chebulinic acid	Inhibits ferroptosis through ROS scavenging and iron chelation	Bone marrow-derived mesenchymal stem cells isolated from SD rats	/	[207]
Chicoric acid	Activates Nrf2/HO-1 signaling pathway	/	LPS-induced endometriosis in C57BL/6 mice	[208]
	Possible ferroptosis inhibitor thanks to ability to protect against oxidative damage, reduces ROS and MDA production, increases levels of GSH and SOD, and promotes expression of Nrf2 and HO-1	/	Acute lung injury model in male BALB/c mice induced by LPS	[133]
Chlorogenic acid	Upregulates expression of GPX4, SLC7A11, and SLC3A2	Primary cortical neurons isolated from mice	Hypoxic-ischemic brain injury model in neonatal C57BL/6J mice	[209]
	Decreases Fe ^2+^ and MDA accumulation and increased GSH, GPX, GST, and CAT	/	Triptolide-induced multi-organ injury in Kunming mice	[210]
	Upregulates GPX4, GSH, and NADPH; the proposed mechanism is the activation of the IL6/JAK2/STAT3 pathway which leads to reduced production of hepcidin and enhanced expression of FPN1 in the duodenum	/	Chronic stress-induced duodenal ferroptosis model in Wistar rats	[211]
Chrysin	Upregulates expression of SLC7A11 and GPX4, and downregulates expression of ACSL4, TfR1 and COX2	/	tMCAO-induced cerebral ischemia/reperfusion model in SD rats	[212]
Chrysophanol	Inhibits ferroptosis through upregulated expression of GPX4	PC12 cells	Alzheimer’s disease model in SD rats	[84]
Corilagin	Downregulates expression of ACSL4, upregulates expression of GPX4, and inhibits NCOA4-mediated ferritinophagy	/	Intestinal ischemia/reperfusion injury model in C57BL/6J mice	[213]
Curculigoside	Ferroptosis was characterized by iron accumulation, depletion of GSH, ROS, and MDA production with decreased expression of SOD and GPX4, which were attenuated by curculigoside	IEC-6 cells	Model of ulcerative colitis in C57BL/6J mice	[214]
Curcumin	Curcumin-OM-MSCs upregulates expression of GPX4, SLC7A11 and FTH1, and downregulates expression ACSL4	Primary cortical neurons isolated from SD rats	Collagenase IV-induced intracerebral hemorrhage model in SD rats	[50]
	Downregulates ACSL4 and upregulates GPX4	/	Ischemia/reperfusion injury model in Wistar rats	[215]
	Inhibits ferritinophagy through the NCOA4 pathway and activates the Nrf2 pathway	/	AFB1-induced kidney nephrotoxicity in ducks	[45]
	Promotes expression of Nrf2, GPX4, and HO-1	BRL-3A cells	Hepatocellular degeneration model in TX mice	[216]
	Increases expression of SLC7A11 and GPX4 and reduces expression of ACSL4 and TfR1	/	Ligature wire-induced periodontitis model in C57/BL mice	[217]
	Upregulates expression of Nrf2, GPX4, SLC7A11, HO-1, NQO-1, CAT, SOD, FPN1, FTH1, downregulates expression of Keap1, NCOA4, ACSL4, PTG2, TfR1, p53	/	NH_4_Cl-inuced ammonia stress in Gibel carp	[218]
	Increases expression of SLC7A11, GPX4 and FTH1 through activation of Nrf2 pathway	Primary chondrocytes isolated from BALB/C mice	Erastin-induced knee ferroptosis in BALB/C mice	[219]
	Can inhibit multiple cell death mechanisms induced by antimalarial drug mefloquine, including ferroptosis by inhibition of lipid peroxidation	/	Swiss-strain mice infected with Plasmodium berghei	[220]
	Enhances nuclear translocation of Nrf2 and enhances expression of GPX4 and HO-1	H9c2 cells	Streptozotocin-induced diabetes model in New Zealand rabbits	[82]
	Mitigated production of ROS, depletion of GSH, increased levels of iron and MDA, downregulation of SLC7A11, GPX, FTH1, and upregulation of TfR induced by cigarette smoke	BEAS-2B cells	Lung injury model induced by cigarette smoke in Sprague-Dawley rats	[221]
	Inhibition of generation of ROS and upregulation of Nrf2/HO-1 pathway	HT22 cells	Intracerebral hemorrhage model in C57BL/6 mice	[222]
	Upregulation of expression of HO-1	Proximal murine tubular epithelial cells (MCTs)	Rhabdomyolysis in C57BL/6 mice	[223]
	Iron chelation, prevention of GSH depletion, lipid peroxidation, and GPX4 inactivation	MIN6 cells	/	[224]
	Potential ferroptosis inhibitor, reduces lipid peroxidation and preserves the activity of CAT, SOD, and GPX	Primary cardiomyocytes from neonatal Sprague-Dawley rats	Doxorubicin-induced cardiotoxicity in Kun-Ming mice	[225]
Cyanidine-3-glucoside	Decreases levels of Fe^2+^, 4-HNE, MDA, decreases expression of ACSL4, and increases expression of GPX4. AMPK pathway is essential for anti-ferroptosis activity	HK-2 and NRK-52E cells	Renal ischemia/reperfusion injury model in C57BL/6 mice	[226]
	Downregulation of NCOA4 and TfR, and upregulation of GPX4 and FTH1	H9c2 cells	Ischemia/reperfusion injury model in Sprague-Dawley rats	[7]
Cynarin	Alleviates iron accumulation and inhibits ROS production and lipid peroxidation, and upregulates expression of GPX4 and Nrf2	Primary rat nucleus pulpous cells	Caudal intervertebral disc puncture model in SD rats	[227]
Daidzein	Activates Nrf2/SLC7A11/GPX4 pathway	Primary hepatocytes	APAP-induced hepatotoxicity in C57BL/6 mice	[228]
Dihydromyricetin	Inhibits ferroptosis through LCN2/Xct- axis	/	Collagenase-induced intracerebral hemorrhage model in C57BL/6 mice	[229]
	Alleviates iron accumulation, inhibits ROS production, decreases expression of ACSL4, increases expression of GPX4, and inhibits p-JNK inflammatory cytokine pathway	/	High-fat-diet- and streptozotocin-induced diabetes in SD rats	[83]
	Activates Nrf2 pathway, and upregulates expression of SOD, CAT, GCLC, GCLM, GPX4	HK-2 cells	Cisplatin-induced acute kidney injury in C57BL/6 mice	[230]
	Inhibits lipid peroxidation, upregulates expression of GPX4, and downregulates ACSL4	HT22 cells	MCAO-induced cerebral ischemia/reperfusion injury model in SD rats	[231]
	Possible ferroptosis inhibitor, reduces the production of MDA, increases levels of GSH and SOD, and activates Nrf2/HO-1 pathway	HT-22 cells	/	[232]
Dihydroquercetin	Lowers levels of MDA and ROS, increases RNA and protein levels of SLC7A11 and GPX4, and increases levels of Nrf2 in a dose-dependent manner	HBE cells	Cigarette smoke-induced chronic obstructive pulmonary disease in BALB/c mice	[233]
	Upregulates expression of GPX4, FTH1 and NCOA4, downregulates LC3, and inhibits ferritinophagy	HBE and MRC-5 cells	Silica-induced lung fibrosis in C57BL/6 mice	[234]
3,4-dihydroxyphenyletyl alcohol glycoside	Decreases levels of ROS and MDA, increases the level of GSH, restores the activity of CAT and SOD, promotes expression of GPX4, and inhibits expression of HO-1, ERK, NLRP3, Caspase1, and Gasdermine-D	AML12 cells	APAP-induced acute liver failure model in C57BL/6 mice	[235]
Diosmetin	Activates SIRT1/Nrf2 pathway	/	S. aureus-induced mastitis in BALB/c mice	[236]
Echinatin	Increases expression of GPX4, SLC7A11, SLC3A2, FTH1, FTL, GCLC ang GCMC, activates Nrf2 pathway	Primary human umbilical artery smooth muscle cells	Vascular stiffening, 5/6 nephrectomy, and atherosclerotic mouse models in C57BL/6 mice	[237]
	Activates Nrf2 pathway	Primary rat hippocampal neurons	Sevoflurane-induced neurotoxicity in SD rats	[90]
Eleutheroside B	Upregulates expression of GPX4, SLC7A11, FTH1, HO-1, and downregulates expression of TfR1 and COX2, activates Nrf2 pathway	/	SD rats exposed to hypobaric hypoxia	[238]
Emodin	Activates Nrf2/SLC7A11/GPX4 axis	HK-2 cells	Streptozotocin-induced diabetes in SD rats	[239]
Engeletin	Activates Keap1/Nrf2 pathway	Primary BMSCs from SD rats	/	[240]
(-)-epigalocatechin-3-gallate	Upregulates expression of GPX4, and downregulates expression of ACSL4 and COX2	L-02 cells	High-fat-diet-induced hepatic lipotoxicity in C57BL/6 mice	[126]
	Acts on STAT1/SLC7A11 pathway	A549 and RAW 264.7 cells	Urethane-induced lung cancer in C57BL/6 mice	[241]
	Upregulates Nrf2/GPX4 axis	Primary mouse hepatocytes	High-iron-diet-induced iron overload in C57BL/6 mice	[242]
	Acts on miR-450b-5p/ACSL4 axis	HL-1 cells	Acute myocardial infarction model in C57BL/6 mice	[243]
	Activates Nrf2/HO-1 pathway	NRK-52E, HK-2, mTEC and AML-12 cells	Gentamicin-induced nephrotoxicity in SD rats	[39]
	Reduces iron accumulation, oxidative stress, and abnormal lipid metabolism	H9c2 cells and neonatal rat cardiomyocytes	Doxorubicin-induced cardiotoxicity in C57BL/6 mice	[244]
	Upregulates expression of GPX4 and FTH1, downregulates expression of ACSL4 and COX2	Primary cerebellar granule	Spinal cord injury model in rats	[245]
	Reduces production of ROS, and increases expression of GPX4 and SLC7A11, these effects are Nrf2 dependent, and Nrf2 inhibitors abolish EGCG protective effect against ferroptosis	HIEC line	Radiation-induced intestinal injury model in C57BL/6J mice	[246]
	Iron chelation, prevention of GSH depletion, lipid peroxidation, and GPX4 inactivation	MIN6 cells	/	[224]
Eriodictyol	Inhibits ferroptosis through VDR-mediated activation of the Nrf2/HO-1 pathway	HT-22 hippocampal cells	Alzheimer’s disease model in APPswe/PS_1_E_9_ transgenic mice	[89]
	Possible ferroptosis inhibitor, reduces the production of ROS and MDA, restores activity of SOD and CAT, increases levels of GSH, activates Nrf2/HO-1 pathway	BV-2 cells	LPS triggered oxidative stress in C57BL/6 mice	[88]
	Possible inhibitor of ferroptosis, upregulates SOD, CAT, and GPX, and activates Nrf2/HO-1 axis	RGC-5 cells	/	[247]
Farrerol	Upregulates expression of Nrf2, GPX4, SLC7A11 and HO-1	/	Hypoxic-ischemic encephalopathy in SD rats	[248]
	Balances iron metabolism and promotes expression of GPX4 and SLC7A11	Primary tenocytes isolated from rats	Collagenase-induced tendinopathy in SD rats	[249]
Ferulic acid	Activates Nrf2/GPX4 pathway	HT-22 cells	High-fat-diet-induced cognitive impairment in C57BL/6 mice	[84]
	Activates Nrf2/HO-1 pathway	MLE-12 cells	Acute lung injury model in BALB/c mice	[134]
	Activates Nrf2 signaling pathway	MIN6 cells	/	[250]
	Upregulates expression of GPX4 and AMPKα2	/	Myocardial ischemia/reperfusion injury model in SD rats	[251]
Fisetin	Downregulates expression of ACSL4 and COX2, and upregulates expression of GPX4	TCMK-1 cells	Chronic kidney disease model in mice	[252]
	Reduces levels of ROS and MDA, and increases the level of GSH, upregulates SIRT1, Nrf2, GPX4, HO-1, and FTH1	H9c2 cells	Doxorubicin-induced cardiomyopathy in Waster rats	[253]
Formononetin	Enhances expression of SLC7A11 and GPX4, promotes nuclear translocation of Nrf2, and blocks nuclear translocation of Smad3 and ATF3	Primary mouse renal tubular epithelial cells	Chronic kidney disease model in C57BL/6 mice	[254]
Fraxetin	Promotes expression of GPX4 and SLC7A11, and inhibits expression of NCOA4	MLE-12 cells	Bleomycin-induced pulmonary fibrosis in C57BL/6 mice	[255]
	Activates AKT/Nrf2/HO-1 pathway	H9c2 cells	Myocardial infarction model in Wistar rats	[256]
Galangin	Upregulates expression of GPX4, FTH1, and SLC7A11 through activation of Nrf2 pathway	Primary rat cardio-myocytes	Myocardial ischemia/reperfusion injury model in C57BL/6 mice	[108]
	Activates PI3K/AKT/CREB pathway	HT1080 cells	Ischemia/reperfusion injury model in C57BL/6 mice	[109]
	Upregulates expression of SLC7A11 and GPX4	Hippocampal neurons culture	Cerebral ischemia-reperfusion injury model in gerbils	[44]
Gallic acid	Inhibits ferroptosis through modulation of the P2X7-ROS signaling pathway	Primary microglial culture isolated from neonatal SD rats	Chronic comorbid injury and chronic unpredictable mild stimulation model in Sprague-Dawley rats	[257]
Gastrodin	Acts on SIRT1/FOXO3A/GPX4 pathway	HK-2 cells	Cisplatin-induced nephrotoxicity in C57BL/6 mice	[258]
	Activates Nrf2/GPX4 signaling pathway	HT22 cells	BCCAO-induced vascular dementia model in SD rats	[91]
	Upregulation of oxidative defense system through Nrf2/HO-1 axis	HT22 cells	/	[92]
	Decreases levels of MDA and ROS, raises the level of GSH, and increases GPX activity, upregulates expression of Nrf2, GPX, HO-1, and FPN1	C6 cell line	/	[47]
	Potential ferroptosis inhibitor, upregulates expression of Nrf2 and HO-1	Liver sinusoidal endothelial cells isolated from C57BL/6 mouses	/	[259]
Geraniin	Iron chelator and ROS scavenger	bmMSC isolated from rat femur and tibia	/	[260]
Gingerenone A	Activates Nrf2/GPX4 signaling pathway	HepG2 cells	Dextran sodium sulphate-induced secondary liver injury in C57 mice	[261]
6-Gingerol	Decreases iron and MDA levels, increases the activity of SOD, inhibits expression of FACL4, promotes expression of GPX4, and activates Nrf2/HO-1 axis	H9c2 cells	Streptozotocin and high-fat-diet-induced diabetes mellitus model in C57BL/6 mice	[262]
8-Gingerol	Upregulates expression of GPX4 and downregulates expression of LOX15	HT22 cells	Spinal cord injury model in SD rats	[263]
Glabridin	Upregulates expression of GPX4, SLC7A11, SLC3A2 and downregulates expression of TfR1	NRK-52E cells	High-fat-diet- and streptozotocin-induced diabetes in SD rats	[81]
Gossypol acetic acid	Decreases levels of Fe^2+^ and ROS, inhibits lipid peroxidation, upregulates GPX4	H9c2 cells	Cardiac ischemia/reperfusion injury model in Sprague-Dawley rats	[264]
Hesperidin	Activates Nrf2 signaling pathway	Primary human nucleus pulposus cells	Disc degeneration model in mice	[265]
Honokiol	Upregulates expression of GPX4 and SLC7A11 by activating Nrf2 pathway	RSC96 cells	Streptozotocin-induced diabetes in SD rats	[266]
Hydroxysafflor yellow A	Activates HIF-1α/SLC7A11/GPX4 Signaling pathway	H9c2 cells	Myocardial ischemia/reperfusion injury model in C57BL/6 mice	[267]
	Upregulates expression of SLC7A11 and GPX4, downregulates expression of ACSL4, downregulates expression of miR-429 which additionally upregulates expression of SLC7A11	HUVECs	High-fat-diet- and streptozotocin-induced diabetes in ApoE^−/−^ C57BL/6 mice	[268]
	Reduces ROS, 4-HNE, and MDA production, reduces Fe^2+^ levels, increases GSH/GSSG ratio, and upregulates expression of GPX4 and SLC7A1	PC12 cells	/	[191]
Hyperjaponol J	Suppresses RSL-3-induced ferroptosis	HT22 cells	/	[269]
Hyperjaponol K	Suppresses RSL-3-induced ferroptosis	HT22 cells	/	[269]
Icariin	Activates Nrf2/SLC7A11/GPX4 pathway	/	Methionine choline-deficient-diet-induced nonalcoholic steatohepatitis in C57BL/6J mice	[270]
	Activates SIRT1/Nrf2/HO-1 signaling pathway	HL-1 cells	Excessive-ethanol-treated C57BL/6 mice	[271]
	Upregulates expression of GPX4 and FTH1	HUVECs	Atherosclerosis model in ApoE^−/−^ C57BL/6 mice	[272]
	Alleviates iron accumulation and lipid peroxidation	/	Alzheimer’s disease model in C57BL/6J mice	[93]
	Activates Nrf2/HO-1 signaling pathway	Primary endplate chondrocytes	Intervertebral disc degeneration model in C57BL/6 mice	[273]
	Decreases level of Fe^2+^, upregulates Nrf2, GPX4, HO-1, and downregulates ACSL4	H9c2 cells	/	[274]
	Lowers levels of MDA and iron by increasing expression of GPX4, SLC7A11, SLC3A12L, and Nrf2 and decreasing expression of TfR1 and NCOA4	HUM-CELL-0060 cells	/	[275]
Icariside II	Activates Nrf2 signaling pathway	primary astrocytes	MCAO-induced cerebral ischemia/reperfusion injury in mice	[276]
Isoforsythiaside	Activates Nrf2 signaling pathway	HT22 and BV2 cells	Alzheimer’s disease model in mice	[85]
Isoliquiritigenin	Reduces MDA, Fe^2+,^ and NO levels, increases expression of GPX4 and Xct- system, and reduces expression of NCOA4	HK2 cells	LPS-induced acute kidney injury model in C57BL/6 mice	[277]
Isoliquiritin apioside	Inhibits HIF-1α signaling pathway	MLE-2 cells	Intestinal ischemia/reperfusion-induced acute lung injury in C57BL/6 mice	[278]
Isoquercetin	Decreases the production of ROS and MDA, increases the activity of SOD and CAT, and inhibits the NOX4/ROS/NF-κB pathway by induction of Nrf2 nuclear translocation	Primary rat hippocampal neuron cell culture	Cerebral ischemia/reperfusion injury model in Sprague-Dawley rats induced by MCAO/R surgery	[279]
Isorhamnetin	Possible ferroptosis inhibitor, activates Akt/SIRT1/Nrf2/HO-1 signaling pathway	HT22 cells	/	[280]
	Upregulates SIRT1, Nrf2, and HO-1, and downregulates NOX2/4	H9c2 cells	Hypoxia/reoxygenation-induced myocardial injury	[106]
Isorhapontigenin	Inhibits PRDX2/MFN2/ACSL4 signaling pathway	CMECS	Diabetes model in db/db mice	[281]
Kaempferol	Activates Nrf2 signaling pathway	L02 cells	Acetaminophen-induced liver injury in BALB/c mice	[125]
	Activates Nrf2/SLC7A11/GPX4 axis	Primary mouse cortical culture prepared from E16 mouse embryos	/	[120]
Kumatakenin	Prevents iron accumulation through Eno3/IRP1 and inhibits lipid peroxidation	MODE-K cells	DSS-induced acute colitis in C57BL/6 mice	[282]
Licochalcone A	Upregulates expression of GPX4, downregulates expression of ACSL4, inhibits Nrf2/HO-1 axis	H9c2 cells	Ischemia/reperfusion model in SD rats	[283]
Luteolin	Decreases levels of ROS, MDA, and iron, and restores protein levels of GPX4	H9c2 cells and primary cardiomyocytes from neonatal SD rats	Langerdorff perfusion system for ischemia/reperfusion model on isolated hearts from SD rats	[119]
Loureirin C	Activates Nrf2 signaling pathway	Primary cortical neurons and 5H-SY5Y cells	MCAO/R-induced cerebral ischemia/reperfusion injury in C57BL/6 mice	[116]
Methyl ferulic acid	Downregulates expression of ACSL4 and upregulates expression of GPX4 in a NOX4 dependent manner	/	SNI-induced neuropathic pain model in SD rats	[284]
Moracin N	Upregulates expression of GPX4, SLC7A11, CAT, SOD2, NFE2L2, HMOX1, GCLC, and GCLM, and downregulates expression of ACSL4, PTGS2, and FTH1	HT22 cells	/	[285]
Naringenin	Activates Nrf2/HO-1 signaling pathway	BEAS-2B cells	AgNPs-induced lung injury in ICR mice	[135]
	Decreases expression of NOX1, increases expression of GPX4, SLC7A11, FTH, and FPN1	H9C2 cells	Myocardial ischemia/reperfusion injury model in Sprague-Dawley rats	[115]
Naringin	Targeting of P2Y_14_ receptors, upregulation of Nrf2/GPX4 pathway	/	Streptozotocin-induced diabetic cardiac autonomic neuropathy model in Sprague-Dawley rats	[77]
Nobiletin	Modulates p53/SLC7A11 axis	MLE-12 cells	Heatstroke-induced acute lung injury model in C57BL/6 mice	[286]
	Downregulates expression of ACSL4 and NCOA4	H9c2 cells	High-fat-diet- and streptozotocin-induced diabetes model in SD rats	[287]
	Activates Nrf2/HO-1 signaling pathway	/	Sepsis-associated acute liver injury in C57BL/6 mice	[122]
	Ameliorates oxidative stress and ferroptosis through modulation of GPX4 and FTH1	/	Renal injury model in C57BL/6J mice	[288]
Phlorizin	Alleviates iron overload and lipid peroxidation, promotes expression of GPX4, and decreases expression of FTH1 and FTL1	Primary mouse colonic lamina propria cells	Dextran sulphate sodium-induced colitis in C57BL/6J mice	[289]
Piceatannol	Antioxidant activity	bmMSCs	/	[193]
Pinocembrin	Inhibits ferroptosis through activation of Nrf2 pathway	Primary chondrocytes isolated from C57BL/6 mice	Surgery-induced intervertebral disc degeneration model in C57BL/6 mice	[290]
Polydatin	Reduces levels of iron, ROS, and MDA, increases the level of GSH, and GPX4 activity	HK-2 cells	Cisplatin-induced acute kidney injury model in C57BL/6 mice	[291]
	Increase in GPX4 activity and decreased level of MDA	Neuro2A cells	Traumatic brain injury in male C57BL/6 mice	[100]
Proanthocyanidins	Activates Nrf2/HO-1 signaling pathway	/	MCAO-induced cerebral ischemia/reperfusion injury in ICR mice	[292]
	Downregulates expression of ACSL4, and upregulates expression of GPX4 and SLC7A11	/	Virus infection model in mice	[293]
	Decreased levels of iron, TBARS, downregulation of ACSL4 and ALOX15B, upregulation of GPX4, Nrf2, and HO-1, and increased level of GSH	/	Spinal cord injury in C57BL/6 mice	[294]
Protocatechualdehyde	Possible inhibitor of ferroptosis thanks to the ability to decrease the production of ROS, 4-HNE, and 8-OHdG, and activate the PKCε/Nrf2/HO-1 signaling pathway	SH-SY5Y cells	Focal cerebral ischemia model in Sprague-Dawley rats induced by MCAO	[295]
Protocatechuic acid	Alleviates iron accumulation and lipid peroxidation and promotes expression of GPX4	Caco-2 cells	Ulcerative colitis model in C57BL/6 mice	[296]
Pterostilbene	Activates Nrf2 signaling pathway	/	Diquat-induced intestinal damage in broiler chicks	[297]
	Promotes expression of Nrf2, GPX4, and HO-1	COV434 and KGN cells	/	[298]
Puerarin	Inhibits lipid peroxidation and upregulates expression of GPX4 and FTH1	H9c2 cells	Ischemia/reperfusion injury model in C57BL/6 mice	[49]
	Downregulates expression of ACSL4, and upregulates expression of GPX4 and FSP1	HK-2 cells	Ischemia/reperfusion injury model in SD rats	[299]
	Upregulates expression of Nrf2, GPX4, SOD, HO-, pAMPK, and PGC1α, and downregulates expression of ACSL4	/	Subarachnoid hemorrhage model in SD rats	[101]
	Upregulates expression of GPX4 and FTH1, and downregulates ACSL4 and TfR1	/	LPS-induced myocardial injury in SD rats	[300]
	Reduces levels of ROS, MDA, and iron, increases level of GSH, increases expression of ACSL7A11, GPX4, FTH1, and decreases expression of NOX1	A549 cells	/	[132]
	Decreases levels of free iron and inhibits lipid peroxidation	H9c2 cells	Heart failure model in Sprague-Dawley rats	[301]
Punicalagin	Potential ferroptosis inhibitor; activates Nrf2/HO-1 signaling pathway	Primary BMSCs	Bone defect model in SD rats	[302]
Quercetin	Activates SIRT1/Nrf2/GPX4 pathway	AT2 cells	LPS-induced lung injury in C57BL/6 mice	[130]
	Activates Nrf2/HO-1 signaling pathway	HK-2 cells	Diabetes model in db/db C57BL/KsJ mice	[78]
	Prevents FTH1 degradation by direct interaction with NCOA4	HepG2 cells	Acrylamide-induced liver injury in C57BL/6J mice	[123]
	Activates SIRT1/p53/SLC7A11 pathway	H9c2 cells	Sepsis-induced cardiomyopathy model in SD rats	[303]
	Inhibits lipid peroxidation, and upregulates expression of GPX4 and SLC7A11	RAW 246.7 cells	LPS/ovalbumin-induced neutrophilic asthma in C57BL/6 mice	[304]
	Upregulates expression of GPX4 and PGS2, and downregulates expression of Tf and Id2	Primary oligodendrocyte progenitor cells	Spinal cord injury model in C57BL/6 mice	[305]
	Decreases Fe^2+^ and MDA accumulation, and increases GSH, GPX, GST, and CAT	/	Triptolide-induced multi-organ injury in Kunming mice	[210]
	Upregulates expression of GPX4, SLC7A11, FTH1, FPN1, FSP1, and downregulates expression of ACSL4 and TfR1	/	Deoxynivalenol-induced intestinal damage in BALB/c mice	[306]
	Activates Nrf2/HO-1 pathway	HT22 cells	/	[307]
	Activates Nrf2 signaling pathway	M17, PC12 and SH-SY5Y cells	MPTP-induced neurotoxicity in C57BL/6 mice	[95]
	Activates SIRT1/Nrf2/GPX4/SLC7A11 signaling pathway	HT22 cells	Kainic acid-induced seizures in C57BL/6J mice	[94]
	Promotes TFEB-dependent degradation of ferritin	MCF-7 and MDA-MB-231 cells	/	[72]
	Activates Nrf2 signaling pathway	Primary BMSCs	/	[308]
	Decreases levels of MDA, and increases the level of GSH and GPX4, inhibits ferroptosis through repression of activation transcription factor 3 (ATF3) and repression of HO-1	NRK-52E and HK-2 cells	Acute kidney injury model in C57BL/6J mice induced by renal ischemia/reperfusion or folic acid	[309]
	Lowers levels of ROS and MDA, increases the level of GSH, restores the activity of SOD, and normalizes protein levels of Xct-, GPX4, and VDAC2	INS-1 cells	Type 2 diabetes mellitus model in C57BL/6J mice induced by high-fat diet and streptozotocin	[310]
Resveratrol	Upregulates expression of Nrf2, GPX4, FTH1, and NQO1, and downregulates expression of TfR1 and p53	H9c2 cells	5-FU-induced cardiotoxicity in C57BL/6J mice	[311]
	Activates SLC7A11/GPX4 axis	MLOY4 cells	Diabetes periodontitis model in C57BL/6 mice	[79]
	Upregulates expression of Nrf2, GPX4, SLC7A11, and FTH1	/	High-intensity-exercise-training-induced intestinal damage in Kunming mice	[312]
	Activates SIRT1/p53 signaling pathway	HiPSCs	Heart failure model in C57BL/6J mice	[313]
	Activates SIRT3/FoxO3a pathway, promotes expression of GPX4 and FTH1, downregulates expression of ACSL4	Caco-2 cells	Intestinal ischemia/reperfusion injury model in C57BL/6 mice	[112]
	Activates Nrf2/Keap1 signaling pathway	BEAS-2B cells	/	[314]
	Activates Nrf2/HO-1 pathway	HT22 cells	/	[307]
	Activates SLC7A11/GPX4 pathway	HepG2 cells	/	[315]
	Upregulates expression of GPX4 and FTH1, and downregulates expression of TfR1	H9c2 cells	Myocardial ischemia/reperfusion injury model in SD rats	[111]
	Upregulates expression of GPX4 and SLC7A11	H9c2 cells	Myocardial infarction model in SD rats	[316]
	Alleviates iron accumulation, inhibits lipid peroxidation, and raises GSH levels	Primary cardiomyocytes	LPS-induced endotoxemia model in C57BL/6 mice	[317]
	Upregulates SIRT1/Nrf2 signaling pathway	H9c2 cells	Sepsis-induced cardiomyopathy model in SD rats	[318]
	Decreased levels of iron and ROS, and increased levels of GSH, decreased expression of ACSL4, increased expression of GPX4 and Ferritin	Primary cortical neurons	Focal ischemic brain damage model in SD rats induced by middle cerebral artery occlusion/reperfusion	[110]
	Decreases level of MDA, increases level of GSH, downregulates expression of ACSL4 and COX2, and upregulates expression of GPX4	MIN6 cells	/	[319]
	Decreases levels of iron, ROS and MDA, increases GSH, increases expression of GPX4, FPN1, Nrf2 and SLC7A11, and downregulates STAT1 and Keap1	BV2 cells	/	[180]
Rhein	Activates Nrf2/SLC7A11/GPX4 pathway	HT22 cells	MCAO-induced cerebral ischemia/reperfusion injury in SD rats	[113]
	Downregulates expression of ACSL4 and TfR1, and upregulates expression of GPX4 and SLC7A11 through modulation of Rac1/NOX1/β-Catenin Axis	MPC5 cells	Diabetic nephropathy in C57BL/6J mice	[80]
Rutin	Decreases Fe ^2+^ and MDA accumulation and increased GSH, GPX, GST, and CAT	/	Triptolide-induced multi-organ injury in Kunming mice	[210]
	Activates Nrf2/HO-1 signaling pathway	Hen ovary follicle tissue culture	/	[320]
Salidroside	Promotes expression of GPX4, SOD1 and SOD2, and downregulates expression of ACSL4 by modulating PI3K/AKT signaling pathway	NRK cells	Renal ischemia/reperfusion injury model in SD rats	[321]
	Inhibits endoplasmic reticulum oxidative stress-related ferroptosis through activation of AMPK/SIRT1 pathway	AML12 cells	APAP-induced acute liver injury in C57BL/6J mice	[124]
	Activates Nrf2/GPX4 pathway	/	Alzheimer’s disease model in SAMP8 mice	[96]
	Inhibits lipid peroxidation and promotes expression of GPX4	C2C12 cells, HUVECs and MOVAS cells	Diabetic hindlimb ischemia model in C57BL/6 mice	[322]
	Activates Nrf2/SLC7A11/GPX4 pathway	MLE-12 and RAW 264.7 cells	Lung ischemia/reperfusion injury in C57BL/6 mice	[323]
	Decreased iron concentration, inhibition of lipid peroxidation and production of 4-HNE, and increased expression of GPX4	H9c2 cells	Doxorubicin-induced cardiomyopathy in C57BL/6 mice	[324]
	Inhibits IL-17A mediated ferroptosis through modulation of Act1/TRAF6/p38 MAPK pathway	/	Hyperoxia-induced acute lung injury in KM mice	[131]
	Promotes expression of GPX4, SLC7A11, FPN1 and FTH1, downregulates expression of ACSL4 and TfR1	/	Aging-related renal fibrosis in SAMP8 mice	[325]
	Activates Nrf2/HO-1 signaling pathway	HT22 cells	Alzheimer’s disease model in mice	[97]
Salvianolate	Possible ferroptosis inhibitor, activates Keap1/Nrf2/HO-1 signaling pathway	Primary renal tubular epithelial cells	Renal ischemia/reperfusion injury in C57BL/6J mice	[326]
Salvianolic acid A	Modulates HIF-2α/DUOX1/GPX4 pathway	HK-2 cells	NaAsO_2_-induced kidney injury in C57BL/6J mice	[327]
	Downregulates expression of ACSL4 and upregulates expression of GPX4 and SLC7A11 and modulates iron metabolism	661W cells	Iron overload model in Kunming mice	[328]
Salvianolic acid B	Upregulates expression of GPX4 and FTH1, downregulates expression of TfR1	H9c2 cells	Myocardial ischemia/reperfusion injury model in SD rats	[329]
	Activates Nrf2 signaling pathway	/	Myocardial infarction model in SD rats	[330]
	Potential ferroptosis inhibitor, reduces MDA and H2O2 production, increases the level of GSH, increases the activity of GPX and SOD, and increases expression of Nrf2, HO-1, and NQO-1	Primary cortical neurons	Subarachnoid hemorrhage model in Sprague-Dawley rats and C57BL/6 mice	[331]
Scutellarein	Promotes expression of GPX4, and prevents overexpression of HO-1	BEAS-2B cells	LPS/cigarette smoke-induced chronic obstructive pulmonary disease model in C57BL/6 mice	[332]
Sennoside A	Alleviates iron accumulation, inhibits lipid peroxidation, and raises GSH levels	BV2 cells	Alzheimer’s disease model in mice	[87]
Sesamin	Reduces production of MDA and iron concentration, increases the level of GSH, and activity of SOD, GPX4, increases expression of FPN1 and TfR1, and inhibits expression of FTH1 and FTL	/	PM2.5 induced cardiovascular injury in Sprague-Dawley rats	[333]
Silibinin	Downregulates expression of p53 and upregulates expression of SLC7A11 and GPX4	HT22 cells	Streptozotocin-induced neurotoxicity in SD rats	[99]
	Upregulates expression of GPX4 and FSP1, downregulates expression COX2, activates PINK1-dependent mitophagy	INS-1 cells	/	[334]
Suberosin	Downregulates expression of ACSL4, LOX, LPCAT3, and upregulates expression of GPX4	/	Streptozotocin-induced diabetes in SD rats	[335]
Syringic acid	Activates Nrf2/HO-1/SLC7A11 pathway	C2C12 cells	Femoral artery ischemia/reperfusion injury in C57BL/6 mice	[336]
Tannic acid	Upregulates expression of GPX4, SLC7A11 and ferritin, downregulates expression of ACSL4, TfR1, COX2, LOX, and p53	/	T2-toxin treated C57BL/6J mice	[337]
Tectorigenin	Downregulates expression of ACSL4 and upregulates expression of GPX4	/	LPS-treated C57BL/6 mice	[338]
	Upregulates expression of SLC7A11, GPX4, and downregulates expression of NOX4 and activation of Smad3	Mouse primary renal tubular epithelia cells	Unilateral ureteral obstruction model in C57BL/6 mice	[339]
Tetrahydroxy stilbene glycoside	Activates GSH/GPX4/ROS and Keap1/Nrf2/ARE pathways	/	Alzheimer’s disease model in APP/PS1 mice	[98]
Theaflavin-3,3′-Digallate	Upregulates expression of Nrf2, GPX4, FTH1 and HO-1	Primary culture of human chondrocytes	Osteoarthritis model in SD rats	[340]
Thonningianin A	Activates Nrf2/HO-1 signaling pathway	SH-SY5Y cells	6-hydroxydopamine treated zebrafish	[43]
Trilobatin	Activates Nrf2/HO-1/GPX4 pathway	/	Exhaustive exercise-induced fatigue in C57BL/6 mice	[341]
Umbelliferone	Downregulation of ACLS4 and upregulation of GPX4, Nrf2 and HO-1 expression	HK-2 cells	Diabetic nephropathy model in C57BLKS/J db/db and C57BLKS/J db/m mice	[342]
Vitexin	Downregulates expression of Keap1 and TfR1 and upregulates expression of GPX4, SLC7A11, and HO-1	Primary cortical neuron cells	MCAO-induced cerebral ischemia/reperfusion injury in SD rats	[114]
	Activates Keap1/Nrf2/HO-1 pathway	HK2 and NRK-49 F cells	Chronic kidney disease model in C57BL/6J mice	[343]
	Upregulates GPX4/SLC7A11 axis	HK-2 cells	High-fat-diet- and streptozotocin-induced diabetic nephropathy in SD rats	[344]
Wedelolactone	Upregulation of GPX4	Pancreatic acinar cell line AR42J	Tauro-cholate induced acute pancreatitis in Sprague-Dawley rats	[345]
Xanthohumol	Decreased lipid peroxidation, ROS neutralization, iron chelation, reduced levels of ACSL4 and Nrf2, and modulation of GPX4	H9c2 cells	Langendorff hearth perfusion system in rats	[42]

## 4. Summary

Ferroptosis is a new mechanism of regulated cell death which is characterized by the accumulation of ferrous iron, depletion of GSH, and overproduction of ROS through the Fenton reaction. This leads to lipid peroxidation in the cell membrane and eventually to cell death. Ferroptosis has been connected with many diseases and pathologies such as neurodegenerative diseases, ischemia/reperfusion injury, and liver fibrosis. In some of these conditions, the selective initiation of ferroptosis can be beneficial, for example in tumors and liver fibrosis. On the other side, the inhibition of ferroptosis can be beneficial in cases of neurodegenerative diseases for the prevention of loss of neurons and in case of ischemia/reperfusion injury.

Plants have been used in traditional medicine for centuries, and with the development of science, new disciplines have emerged such as rational phytotherapy. Plants produce an abundance of secondary metabolites, such as polyphenols, which have numerous bioactivities such as antioxidant, anti-inflammatory, anti-cancer, neuroprotective, cardioprotective, immunomodulatory, and other properties. The multitude of bioactivities exhibited by polyphenols presents significant potential for the exploration and development of novel medications and therapeutic approaches.

**Figure 10:**
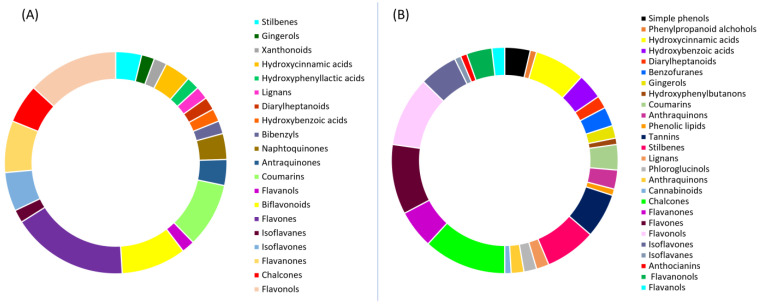
The distribution of polyphenols with pro-ferroptotic (**A**) and anti-ferroptotic (**B**) activity across subclasses.

According to the systematic review carried out within this study, it is evident that the exploration of the connection between ferroptosis and polyphenols, both in vitro and in vivo, is a vibrant ongoing direction for research. It has been confirmed that many polyphenols can modulate ferroptosis, whether through initiation and promotion, or inhibition, which both could have practical implications for health and disease. Yet, as per the review, there is 2.5 times more research on the inhibitory effects of polyphenols on ferroptosis compared to their potential to induce it. This study encompasses the results of research on 143 phenolic compounds. In total, 53 compounds showed an ability to induce ferroptosis, and 110 compounds were able to inhibit ferroptosis, and out of those compounds, 20 showed both abilities depending on the model system. In terms of the inhibition and activation of ferroptosis, the greatest number of papers are on the influence of flavonoids (quercetin, baicalein, baicalin, erianin, puerarin), followed by diarylheptanoids (curcumin) and stilbens (resveratrol). Out of 53 compounds with the ability to induce or promote ferroptosis that were included in this review, 63% belong to flavonoids (the most abundant subclasses are flavones—17%, flavonols—13%, bioflavonoids—9%, and flavanones—8%), followed by coumarins (9%). The frequency of compounds with pro-ferroptotic activity across polyphenol subclasses is shown in Figure 10. A similar trend can be seen for phenolic compounds with anti-ferroptotic activity (Figure 10). Out of the 110 examined compounds that were included in this review, 55% were accounted for by flavonoids (chalcones—12%, flavones—10%, flavonols—10%, flavanones—5%, and isoflavones—5%), followed by stilbenes and hydroxycinnamic acids with 7% each, and tannins (6%). From this, it can be seen that flavonoids were the most studied class of polyphenols especially in subclasses that are not so common in plants such as isoflavones and chalcones. Biflavonoids are present among pro-ferroptotic compounds but are absent from anti-ferroptotic compounds which can be of interest for further research of their mechanisms, especially for diseases where ferroptosis induction would be favorable. Additionally, quercetin and rutin are one of the most studied compounds in this review. When it comes to ferroptosis inhibitions, many stilbenes, cinnamic acids and their derivatives, and tannins have shown promising results. This highlights the importance of research on other classes not just flavonoids (probably highly researched due to their wide-ranging activities and long-lasting use in traditional medicines).

The most extensively studied compounds as inducers of ferroptosis include curcumin, erianin, and shikonin. On the flip side, the most extensively studied inhibitors of ferroptosis include baicalein, curcumin, quercetin, and resveratrol. All the compounds listed are already recognized in natural product research for their beneficial pharmacological properties, and the plants that naturally produce them have been extensively documented in traditional medicine for centuries [37,46,50,60,78,184,316].

Curcumin is a polyphenolic compound derived from the rhizomes of the turmeric plant (*Curcuma longa* L.). It is a natural phenolic pigment that imparts the characteristic yellow color to turmeric. In scientific terms, curcumin is a diarylheptanoid, belonging to the curcuminoid class. It is not surprising that curcumin is so widely investigated regarding its potential to modulate ferroptosis, since it has significant attention in scientific research due to its diverse pharmacological properties, including antioxidant, anti-inflammatory, anti-cancer, and neuroprotective effects. Its versatile biological activities make it a subject of interest in various fields, including pharmacology, medicine, and nutrition. Researchers frequently investigate curcumin’s bioavailability, metabolism, and safety profile to enhance its therapeutic potential. It is essential to note that despite its promising attributes, challenges related to curcumin’s low bioavailability have prompted ongoing efforts to develop formulations that improve its absorption and effectiveness in biological systems, what would also be an issue for ferroptosis modulation in rational therapy [45,50,56,222,225].

Eranin, is a biphenyl compound, which has a historical application as an antipyretic and analgesic agent. It is a constituent of plants from the Dendrobium genus, recognized among the 50 foundational herbs in traditional Chinese medicine. Researchers are particularly interested in eranin due to its demonstrated potential for treating conditions such as inflammation, diabetic nephropathy, retinopathy, psoriasis, and various cancers. Given that ferroptosis is implicated in the development of listed conditions, it come as no surprise that eranin demonstrated potential as a modulator of ferroptosis [37,41,57,66,67].

Furthermore, shikonin is a naphthoquinone derivative obtained from the roots of plants, particularly *Lithospermum erythrorhizon* Siebold & Zucc. 1846. The compound’s distinctive red color makes it easily identifiable. Shikonin and its enantiomeric analogue, alkannin, are prevailing natural lead compounds in the drug discovery and development of anticancer agents. Despite having numerous biological effects, the most important activity reported for shikonin derivatives is their antitumor effect which is exerted through various mechanisms such as the induction of apoptosis and autophagy. The listed body of evidence in this review supports adding ferroptosis to this list [73,74,182,184].

Moreover, both baicalein and quercetin are classified within the flavonoid group of polyphenols, and they are abundantly present in a variety of fruits and vegetables. Numerous studies have substantiated their positive impact on human health, underscoring their potent antioxidant, anti-inflammatory, and anticancer properties. Consequently, it is unsurprising that researchers have shown considerable interest in these compounds, as they demonstrate substantial potential in inhibiting ferroptosis [95,118,197,306].

Wine and grape juice, particularly leaves and skins, are rich sources of resveratrol, a stilbenoid polyphenolic compound. Additionally, resveratrol can be derived from various foods, including peanuts, pistachios, blueberries, strawberries, etc. Research has demonstrated that resveratrol possesses antitumor, antioxidant, anti-inflammatory, and anti-apoptotic effects. This anti-apoptotic effect can be partially explained by its great potential to inhibit ferroptosis [111,175,180,307,319].

Even though polyphenols show promising results for the development of drugs and therapeutics for many diseases, their application can be difficult. Namely, many polyphenols have low bioavailability which can make drug delivery difficult. Curcumin and flavonoid aglycones have high hydrophobicity and are poorly absorbed in the intestine, this can be enhanced through newly developed drug delivery strategies such as the formulation of liposomes or nanoparticles. The next problem with the application is the extensive metabolic transformation that polyphenols can go through in an organism. Hydrophobic compounds such as flavonoid aglycons can be glucuronidated and sulfated which enhances their solubility in body fluids, but at the same time results in their fast excretion through the kidneys. This not only makes it difficult to predict in which state the compounds would be delivered to the target and in which concentration but also poses a question of whether the metabolites will have the same activity as pure compounds. Sometimes metabolites are completely inactive while in other cases metabolites can show greater or different activities. This is a big flaw of in vitro assays on cells, because absorption, bioavailability, and metabolic changes that happen in the body are not encompassed by these experiments. Animal studies, being performed on living organisms, give a better idea about the efficacy of examined compounds and their pharmacokinetics and can be a great stepping stone for the design of new compounds with more desirable properties [6,346,347,348]. Another thing that needs to be taken into consideration when considering the application of polyphenolics as medicines is their potential interaction with other drugs. Polyphenols can bind iron in complexes from which it is not bioavailable or influence the systemic metabolism of iron which needs to be taken into consideration when iron supplementation is being used for the treatment of iron-deficiency anaemia [349]. Another thing that needs to be taken into consideration is the ability to influence the P450 enzyme. P450 is a group of liver enzymes that play a crucial role in the detoxication, metabolism, and removal of different molecules, including drugs. The rate of their activity is crucial for the metabolism and pharmacokinetics of drugs. Polyphenols can lower the activity or inhibit the activity of P450 enzymes, which can result in the accumulation and prolonged activity of drugs, as well as increase the risk of side effects or toxic effects due to higher doses of drugs in the body. On the other hand, some polyphenols can induce P450 activity, which leads to faster metabolism and removal of drugs and can result in low drug efficacy [350]. All this highlights the importance of studying not just bioavailability but also the metabolism and metabolic effects of polyphenolics before their clinical application.

The use of artificial intelligence (AI) and machine learning in different areas has become a hot topic, and interest is still growing. This is due to the various possibilities of applications such as searching for potential targets for therapeutics or design and the development of new drugs. If trained well through machine learning, AI can make this process much easier, faster, and economically affordable because then only the most promising compounds would be synthesized and tested. Recently, there have been some promising results for the use of machine learning to train AI to discern whether the mechanism of cell death was through apoptosis or ferroptosis. This was completed through the synthesis of a database of results obtained in the lab and the use of certain biomarkers for each cell death mechanism [351,352]. Deep learning was also applied to identify new potential targets for treatment of lung cancer through the modulation of ferroptosis [353]. These studies are just stepping stones for future research and showcase the potential and power that AI and machine learning have to offer.

## 5. Conclusions

From the data gathered within this review, it can be summed up that the parameters examined during the evaluation of the potential of polyphenols to promote or inhibit ferroptosis in most cases include the evaluation of levels of GSH, oxidized GSH, MDA, free cellular iron levels, ROS, and the expression of genes and proteins involved in the regulation of ferroptosis, such as GPX4, HO-1, Nrf2, FTH1, and others.

Although there’s been a lot of research on the potential of polyphenols to modulate ferroptosis, very little is known about the influence of plant extracts that contain numerous polyphenols, with some of them possibly possessing antagonistic activities. Additionally, the effects of polyphenols on ferroptosis can vary depending on factors such as concentration and bioavailability. It would be of interest to construct structure–activity relation studies to gain insight into what structural elements of polyphenols are responsible for their desired activities toward ferroptosis, which can open the door to the development of new drugs based on naturally occurring phytochemicals. Also, there is a need for further study of the role that ferroptosis plays in some diseases which would provide additional targets for potential treatments.

According to the author’s opinion, the emerging field of ferroptosis and its modulation by natural compounds like polyphenols presents a fascinating and promising area of research with profound implications for human health and disease, notably for cancer, cardiology, neurology, and transplantation. The intricate interplay between ferroptosis and various physiological processes underscores the complexity of cellular regulation and underscores the potential for innovative therapeutic interventions. Furthermore, the duality of polyphenols in either promoting or inhibiting ferroptosis, as exemplified by compounds like curcumin, highlights the complicated nature and wide range of natural product pharmacology. This complexity underscores the importance of comprehensive research efforts to unravel the precise mechanisms underlying these effects and their potential applications in different disease contexts. As we delve deeper into understanding ferroptosis and its modulation, it becomes increasingly clear that exploring not only the molecular pathways involved but also the practical aspects of delivering these compounds into specific cells is essential for translating research findings into effective clinical interventions. Overall, the study of ferroptosis and its interaction with natural compounds like polyphenols represents an exciting frontier in biomedical research, offering new insights and opportunities for improving human health and combating various diseases.

## Data Availability

No new data were created or analyzed in this study. Data sharing is not applicable to this article.

## Supplementary Materials

The following supporting information can be downloaded at https://www.mdpi.com/article/10.3390/antiox13030334/s1, Table S1: PRISMA 2020 for Abstracts Checklist; Table S2: PRISMA 2020 Checklist.
